# Immunometabolic reprogramming of *Mycobacterium tuberculosis*-responsive memory CD4^+^ T cell subsets is linked to long-term protective immunity

**DOI:** 10.3389/ftubr.2026.1783887

**Published:** 2026-04-10

**Authors:** Vaishnavi Kaipilyawar, Samantha Leong, Arianne Lovey, Lorenzzo L. Stringari, Reynaldo Dietze, Jerrold J. Ellner, Rodrigo Ribeiro-Rodrigues, Padmini Salgame

**Affiliations:** 1 Department of Medicine, Center for Emerging Pathogens, Rutgers New Jersey Medical School, Newark, NJ, United States; 2 Núcleo de Doenças Infecciosas, Universidade Federal do Espirito Santo, Vitória, Brazil; 3 Laboratório Central de Saúde Pública do Estado do Espírito Santo (LACEN-ES), Secretaria de Saúde do Estado do Espírito Santo, Vitória, Brazil

**Keywords:** central memory (T) CM, effector memory (T) EM, immunometabolism, latent TB (LTBI), memory CD4^+^ T cell subsets, stem cell memory (T) SCM, transitional memory (T) TM, tuberculosis

## Abstract

**Introduction:**

Memory CD4^+^ T cells are central to long-term immunity in tuberculosis (TB), yet their functional roles that define their protective capacity remain unclear. Understanding the immune mechanisms that prevent clinical progression from latent TB infection (LTBI) to active TB disease is critical for the development of next-generation vaccines and biomarkers.

**Methods:**

We characterized the transcriptomic, metabolic, and functional programs of Mycobacterium tuberculosis (Mtb) antigen-stimulated peripheral CD4^+^ T stem cell (T-SCM), central (T-CM), transitional (T-TM), and effector (T-EM) memory subsets from individuals with remote LTBI. We utilized a multi-platform validation strategy that integrated RNA-sequencing data with protein-level metabolic profiling using “Met-Flow” cytometry and functional growth restriction assays to link memory CD4^+^ T cell differentiation states to immunometabolism and antimycobacterial function. Finally, we evaluated the immunometabolic profiles of memory CD4^+^ T cell subsets in an independent, longitudinal cohort of Mtb-exposed progressors and non-progressors from Brazil (GSE112104).

**Results:**

We identified a differentiation gradient associated with distinct immunometabolic states. T-SCM and T-CM subsets exhibited elevated mitochondrial activity and oxidative metabolism (fatty acid oxidation), supporting their proliferative capacity. In contrast, T-TM and T-EM subsets underwent glycolytic reprogramming and engaged the pentose phosphate pathway, which fueled enhanced cytokine production and Mtb growth restriction. Importantly, we observed that non-progressors exhibit fatty acid oxidation-driven, stem/central memory-like signatures, while progressors and active TB cases display elevated exhaustion markers, glycolytic reprogramming and pro-inflammatory profiles aligned with disease progression.

**Conclusion:**

Collectively, findings from our proof-of-concept study suggest metabolic state as a key axis connecting Mtb antigen-induced memory T cell differentiation, restimulation-induced transcriptional programming, and durability of immune control. The findings provide the basis for future longitudinal studies to examine the dynamic metabolic and functional modulation in Mtb antigen-specific memory T cell subsets from contained infection to disease progression.

## 1 Introduction

Tuberculosis (TB) continues to be among the world's deadliest infectious diseases, causing morbidity and mortality in millions of people annually (1). Most individuals infected with *Mycobacterium tuberculosis* (Mtb) develop memory immune responses that define latent TB infection (LTBI) with no clinical signs of active TB disease (2). Memory T cells are critical mediators of protection against the progression to active TB, exhibiting Mtb antigen-specific recall responses (3, 4). Hence, sustaining a pool of memory T cells in circulation is essential for continuous immune surveillance and for the rapid initiation of effector functions following antigen re-exposure.

CD4^+^ T cells play a critical role in protective immunity against Mtb, as evidenced by extensive experimental and clinical studies. Their importance is underscored by the increased susceptibility to Mtb infection observed among individuals living with HIV, who experience a loss of CD4^+^ T cells (5–8), potentially impairing the formation of the effector memory response against Mtb (9). Yet, memory CD4^+^ T cell responses against Mtb remain incompletely understood. Previous studies, largely based on CD8^+^ T cells in cancer and viral infection models, have revealed the existence of specialized memory cell subsets capable of differentiation into CCR7^+^ CD62L^+^ central (T-CM) and CCR7^−^ CD62L^−^ effector (T-EM) memory compartments based on tissue homing potential and effector function (10, 11). The circulating memory cell classification scheme has since been expanded by the discovery of other stable stages of memory cell differentiation: “self-renewing” stem cell memory (SCM) and the “effector-like” transitional memory (TM) T cells, based on additional markers including the T cell co-stimulatory marker CD27 and apoptosis-associated Fas receptor CD95 (12–14). Compared to T-EM cells, T-TM cells exhibit greater proliferative capacity, longer telomere lengths, and increased IFNγ production following *in vitro* stimulation (12). T-SCM cells demonstrate superior persistence following antigen loss compared to other memory subsets, potentially playing a role in long-term cellular immunity by acting as the progenitor of memory cells upon antigen re-exposure (13, 15, 16).

Some studies have indicated that the qualitative characteristics of memory T cells induced in individuals with LTBI may govern long-term protection and progression to TB disease (17–20). Specifically, functional capacity, longevity, and the ability to mount recall responses is intricately linked to cellular metabolism (21). For instance, differential expression of key metabolic regulators and rate-limiting enzymes, such as those involved in glycolysis or oxidative phosphorylation and fatty acid metabolism, may reprogram T cells either for rapid proliferation and cytokine production or support long-term survival and self-renewal functions (11, 21, 22). CD4^+^ memory T cells have been reported to exhibit such self-renewing and effector phenotypes similar to those characterized in CD8^+^ memory cells (23–26), but their functional roles in Mtb-specific responses have not been well-studied. Ultimately, a clear understanding of the dynamics of Mtb antigen-responsive memory CD4^+^ T cell subsets, particularly their metabolic underpinnings and recall functions, will be foundational to the design of novel TB vaccines and in identifying risk of disease progression biomarkers.

Therefore, in this study, our goal was to uncover the immune response profiles of Mtb antigen-responsive CD4^+^ memory T cell subsets. We first evaluated the transcriptomes of Mtb antigen-stimulated CD4^+^ memory T cell subsets in peripheral blood mononuclear cells (PBMC) obtained from long-term asymptomatic individuals with LTBI (remote LTBI), and validated these findings at the single-cell protein level using “Met-Flow” cytometry. We then linked these subset-specific responses to functional outcomes, including cytokine production and Mtb growth restriction. Finally, we performed an external population-level concordance analysis using baseline samples from a previously published longitudinal cohort of Mtb-exposed household contacts with known TB outcomes (GSE112104). Concordant with our results, immunometabolic signatures of activated CD4^+^ memory T cell subsets of “non-progressors” differed from individuals who ultimately developed TB “progressors”. Together, these analyses provide an integrated characterization linking memory T cell differentiation, metabolism, and functional Mtb control.

## 2 Methods

### 2.1 Study participants and ethics statement

Healthy asymptomatic donors from Brazil (*n* = 5, males and females) and two independent cohorts of donors from North America (*n* = 4 and *n* = 3, males and females) with remote latent TB infection (LTBI) were recruited into a volunteer study (age range: 25–65 years). Donors had a history of positive PPD and IGRA provided a one-time peripheral blood donation. In addition, LTBI status was confirmed by administration of PPD and measurement of induration (all values ≥ 15 mm). Our study examined biologically male and female individuals with LTBI, and comparable findings were reported across both sexes.

### 2.2 PBMC isolation and *ex vivo* stimulation

Peripheral venous blood was collected from each subject using BD Vacutainer tubes with Sodium Heparin (BD #366480). Peripheral blood mononuclear cells (PBMC) were isolated by Ficoll gradient separation method using Histopaque-1077 (Sigma-Aldrich #10771) in Brazil or using Ficoll-Paque (GE Healthcare #17–5446-52) and SepMate-50 tubes (Stem Cell Technologies #15460) in USA as per manufacturer's protocols. PBMC were cryopreserved using 90% heat-inactivated fetal bovine serum (GIBCO #12657–029, South America origin) and 10% DMSO (Sigma-Aldrich #D2650) for storage in liquid nitrogen and cryoshipment to the United States. Upon flash thawing cells in a 37 °C water bath, PBMC were immediately washed in pre-warmed culture medium, consisting of RPMI 1640 (Corning #15040CM), 10% defined fetal bovine serum (GE Healthcare Life Sciences #SH30070.03, U.S. Origin), 1% L-glutamine (Corning #25005CI), and 1% HEPES buffer (Corning #25-060-CI). Washed cells were re-suspended in 5 ml of culture medium in a lightly capped conical tube for an 8-h rest period in a 37 °C, 7% CO_2_ humidified incubator (27). Rested PBMC were seeded at a density of 5 x 10^6^ cells/ml in a volume of 300 μl/well (1.5 x 10^6^ cells/well), in a 96-well V-bottom, 2 ml deep-well plate (Corning #3960), to facilitate T cell-antigen presenting cell interaction during stimulation with Mtb antigens.

PBMC were stimulated in culture medium containing co-stimulants anti-CD28 (BD #555726; 2 μg/ml) and anti-CD49d (BD #555501; 2 μg/ml) with or without Mtb antigens: 5 μg/ml ESAT6 peptide array (BEI resources #NR-34824), and 5 μg/ml CFP10 peptide array (BEI resources #NR-34825); and labeled as “Mtb-stim” or “Co-stim-only” respectively, and cultured for a 16-h period in a 37 °C 5–7% CO_2_ humidified incubator. Golgi transport inhibitor Brefeldin A (Acros/ThermoFisher 10 μg/ml) was added for the final 4 h of stimulation.

### 2.3 Flow cytometry staining and sorting for RNA sequencing

PBMC were pelleted, washed with FACS (PBS + 2% fetal bovine serum (FBS) and 0.09% sodium azide) buffer and treated with Fc block (BD 564765, 2 μg/ml). Cells were then washed twice prior to surface marker staining for 25 min in the dark at 4 °C using the following fluorescently-conjugated antibodies: APC anti-CD3 (BD #555335), PE-Cy5.5 anti-CD4 (eBioscience #35-0047-42), PE-Cy7 anti-CD8 (BD #560917), AF700 anti-CD45RA (BD #560673), BUV395 anti-CD45RO (BD #564291), BB515 anti-CD95 (BD #564596), PE anti-CCR7 (BD #560765), and PE-Cy5 anti-CD27 (eBioscience #15-0279-42). Surface-stained cells were washed with FACS buffer prior to nuclear staining with DAPI (BD #564907) for 5 min in the dark at room temperature. Stained cells were re-suspended in FACS buffer in preparation for sorting. Memory subsets were sort-purified using BD FACSAria II cell sorter paired with the BD FACSDiva Version 6.1.3 software (BD Biosciences, San Jose, CA). Color compensation was performed using single color-stained BD Anti-Mouse Compensation Particles (BD #552843). User-defined gates were determined based on fluorescence-minus-one staining controls. Cells were processed through the sorter during two sorting rounds (Supplementary Figure S1). In the first round of sorting, live (DAPI-negative), CD3^+^, CD4^+^ lymphocytic cells were isolated. In the second round of sorting, live CD4^+^ memory subsets were defined based on differential surface expression of CD45RA and CD45RO isotypes, CCR7, CD27, and CD95 as has previously been described (10, 12, 13). Cell subtypes were identified as follows: stem cell memory T cells (T-SCM): CD45RA^+^CD45RO^−^CCR7^+^CD27^+^CD95^+^; central memory T cells (T-CM): CD45RA^−^CD45RO^+^CCR7^+^CD27^+^CD95^+^; transitional memory T cells (T-TM): CD45RA^−^CD45RO^+^CCR7^−^CD27^+^CD95^+^; and effector memory T cells (T-EM): CD45RA^−^CD45RO^+^CCR7^−^CD27^−^CD95^+^. Final cell populations were directly sorted into clean microcentrifuge tubes containing sort buffer. Cells were centrifuged, washed and buffer was aspirated to obtain live cell pellets for downstream RNA extraction.

### 2.4 Cell proliferation assay and Met-Flow assay

PBMC from donors were labeled with CFSE dye (Biolegend 423801, 5 μm) before stimulation and staining to track *in-vitro* cell division and quantified via flow cytometry at 72 h. For Metflow assay, five metabolic markers were chosen based on their critical role in metabolic pathways (Supplementary Table S2). Purified metabolic antibodies were purchased from Abcam: CPT1A (ab220789), HK1 (ab233837), G6PD (ab133525) and custom-conjugated to fluorophores using AlexaFluor350 Labeling Kit (ThermoFisher A20180), PacificBlue Labeling Kit (ThermoFisher P30013) and PerCP/Cy5.5^®^ Conjugation Kit-Lightning-Link^®^ (ab102911), respectively. Mitotracker-Red CMXROS (ThermoFisher M46752) was utilized to measure mitochondrial activity. PBMC were stimulated and stained for flow cytometry as previously described, with the addition of the following surface marker antibodies: GLUT-1-APC (BD 566580), OX40-BV605 (Biolegend 350028) and CD69-PacificBlue (Biolegend 310930). For detecting metabolic proteins and cytokines, cells were fixed and permeabilized using Cytofix/Cytoperm (BD) for 20 min at 4 °C, followed by intracellular staining with all other metabolic proteins, IFNγ-BUV737 (BD 612845) and IL-17A-BV786 (Biolegend 512338) in 1X permeabilization buffer (eBioscience) for 40 min in the dark at 4 °C. Cells were washed twice and resuspended in 200 μl FACS buffer until acquisition on BD Fortessa X-20 cytometer at Rutgers.

### 2.5 Mtb growth restriction assay

Sixty milliliter of fresh whole blood was obtained from four volunteer donors with LTBI. The whole blood was then added 1:1 to PBS (Corning #21-031-CV) containing 2% FBS (Hyclone #SH30070.03). Thirty milliliter of Blood + PBS + 2% FBS was then added to Sepmate-50 PBMC isolation tubes (Stem Cell Technologies #15450) containing 15 ml Ficoll-Paque PREMIUM 1.073 (GE Healthcare #17–5446-52). SepMate-50 tubes were then centrifuged at 1,200 x *g* for 10 min at room temperature. The top layer was poured off and washed with 20 ml PBS + 2% FBS and centrifuged at 300 x *g* for 10 min at room temperature. Cells were counted and CD14^+^ monocytes were isolated using EasySep Human CD14 Selection Kit II (Stem Cell Technologies #17858) according to the manufacturer's instructions. CD14^−^ cells were reserved and stained according to the Memory T cell staining and sorting protocol as described in 2.3. CD14^+^ monocytes were washed in 20 ml EasySep Buffer (Stem Cell Technologies #20144), centrifuged at 300 x *g* for 10 min at room temperature and re-suspended in supplemented RPMI media as described above, to achieve a density of 1 x 10^6^ cells per ml. 5 x 10^4^ CD14^+^ monocytes were plated in each well of a white, clear bottom 96-well plate (Corning #3610) and allowed to adhere overnight. Adherent cells were then infected with 5 MOI of luciferase-expressing Mtb Erdman. After 4 h, extracellular Mtb was removed from cells by treating with 200 μg/ml Amikacin and washing thrice with PBS + 2% FBS. Two hundred microliter of RPMI based culture media containing the indicated T memory subsets (no T cells, 1:1 T-SCM, 1:1 T-CM, 1:1 T-TM, or 1:1 T-EM, sorted via FACS from each donor) for co-culture were added to each of the wells. Culture media was refreshed on day 2 of the experiment. Relative Luminescence Units (RLU) as a readout of bacterial burdens were determined via GloMax Luminometer (Promega) by quantifying luminescence on days 0, 1, 3 and 5 post infection. For colony forming units (CFU) assay, cells were lysed and lysates were plated on 7H11 agar plates on days 2 and 4 post infection, and colonies were manually counted after 21 days.

### 2.6 RNA extraction and sequencing

RNA extraction was performed using the Qiagen RNeasy Micro Kit (Qiagen #74004) according to the “Purification of Total RNA from Animal and Human Cells” protocol provided by the manufacturer. Total extracted RNA was shipped to the MedGenome Inc. (Foster City, CA) genomics facility for downstream RNA processing and sequencing. RNAseq libraries were prepared using Illumina TruSeq RNA Sample Preparation Kit v2. In brief, mRNA was enriched via poly (A) tail enrichment, and purified mRNA was fragmented using divalent cation under elevated temperature. RNA fragments were used for first strand cDNA synthesis with reverse transcriptase and random hexamer, followed by second strand synthesis using DNA polymerase I and RNase H. cDNA fragments were then used to prepare Illumina libraries by end repair to generate blunt ends and to add a 5'-phosphate group, A-tailing to add a 3'-A overhang, and ligation to Illumina indexed adaptors. Libraries were assessed for quality control using D1000 ScreenTape prior to sequencing. Sequencing was performed using Illumina HiSeq 2500 v4 chemistry with 100 bp read length in high output mode to generate 50 million total reads per sample.

### 2.7 Data analyses and statistics

#### 2.7.1 RNA sequencing data processing and quality control

Raw FASTQ files were evaluated for quality control, and all sample reads met quality standards with an average Phred quality score of 34.72 ± 0.44, GC percentage of 48.69 ± 0.25, and percentage of reads with Phred quality scores >30 of 90.70 ± 1.91. On average, 32.51 ± 4.98 million reads (all of 100 bp length) per orientation were obtained per sample. Low quality reads and adapters were trimmed using Cutadapt (28), and trimmed reads were mapped to the human genome GRCh37 (hg19) using STAR (29). Expression levels for each gene were counted using HTSeq (30) according to ENSEMBL gene annotations (Ensembl 92, Dec 2017).

#### 2.7.2 Differential expression analysis

For differential gene expression analysis, three factors (donor individual, CD4^+^ memory T cell subset, and stimulation condition) were considered. The Bioconductor DESeq2 package (31), using default settings, was used in R to identify differentially expressed genes (DEGs) between samples stimulated with co-stimulants plus Mtb antigens (Mtb stim) vs. samples receiving co-stimulants only (co-stim) (as described above). In the initial global analysis, DEGs were identified by contrasting expression based on stimulation condition, while considering possible variation due to individual and subset via a multi-factor design (“design = ~ individual + subset + condition”). To identify differential gene expression due to stimulation by each cell subset in isolation, an interaction term approach, as described in “Section 3.3- Interactions” of the DESeq2 manual, was used. In brief, the subset and condition factors were combined into a single factor (subsetgroup) with all possible combinations of the original factors. The analysis design was changed to assess for contrasting DEGs based on this new combined term (“design = ~ individual + subsetgroup”). This method enabled identification of gene expression differences due to stimulation condition for each of the four cell subsets in isolation. A subset of DEGs with an adjusted *p*-value cutoff of < 0.001 was used for downstream pathway and enrichment analyses.

#### 2.7.3 Data visualization

The Bioconductor DESeq2 package (31) was used to normalize raw gene counts via log_2_ normalization using the “rlog” function. Gene expression heatmaps were plotted in R using either the pheatmap package (32) with the RColorBrewer package. Principal components analysis was performed using the “prcomp” function in R, and results were plotted using the ggplot and RColorBrewer packages.

#### 2.7.4 Ingenuity pathway analysis

DEG lists (*p* < 0.001) and log_2_ fold changes obtained for each of the four subsets were used as input for analysis using the “Core Analysis” function of Qiagen's Ingenuity Pathway Analysis (IPA) software (QIAGEN Inc.) using z-score and *p* < 0.01 to identify and visualize the activation/inhibition of enriched canonical pathways. The four resulting Core Analysis results were compared using the IPA “Comparison Analysis” function. IPA core analyses and a comparison analysis were also performed on the four DEG lists containing unique (non-overlapping) DEGs for each subset. Data were plotted using GraphPad Prism Software v10.

#### 2.7.5 NanoString assay and gene ontology pathway analysis

The nCounter^®^ Human Metabolic Pathways Panel CodeSet (NanoString Technologies, Seattle, WA, USA) profiling 768 genes involved in core immunometabolism, and metabolic processes was used to measure gene expression in RNA samples from sorted T-memory cell subsets. Following the manufacturer's instructions, 50 ng of RNA from each sample was hybridized with the CodeSet for 18 h, and transcripts were quantitated using the nCounter^®^ SPRINT Profiler (NanoString Technologies, Seattle, WA, USA) at Rutgers. Raw data QC and normalization was performed using NanoString nSolver^®^ version 4 software (NanoString Technologies, Seattle, WA, USA) with background subtraction. PCA analysis was conducted using the ClustVis web tool (33) with the following parameters: ln (x+1) data transformation, unit variance scaling and SVD with imputation. Gene Ontology (GO) pathway analysis of highly expressed genes (log_2_ expression >2.5) in the four subsets was performed with ShinyGO 0.82 (34), using the “GO Biological Process” database with a minimum pathway size of 200 and FDR cut-off of 0.05. Data were visualized using lollipop charts showing fold enrichment.

#### 2.7.7 Cellular deconvolution analysis for GSE112104 cohort

Bulk RNAseq data files from PBMC samples of GSE112104 (16 TB progressors (6 “early” and 10 “late”) and 21 non-progressors) were subjected to cellular deconvolution analysis using CIBERSORTx (35). Files were accessed, pre-processed to format into mixture files and to possess matching HUGO gene identifiers. Data were then mapped to the LM22 dataset (36) comprising a signature matrix file consisting of 547 genes that accurately distinguish 22 mature human hematopoietic populations isolated from peripheral blood or *in vitro* culture conditions, including seven T cell types, naïve and memory B cells, plasma cells, NK cells, and myeloid subsets. Cell expression deconvolution analyses were conducted to compare cell fractions of TB progressors and non-progressors, using a cut-off of *p* < 0.05 and performing 1,000 permutations. Differentially expressed genes of all CD4^+^ memory subsets were exported, and comparison analysis was conducted and visualized using Gene Set Enrichment Analysis of key genes representative of immunometabolic pathways. Data were visualized using GraphPad Prism v10 and R version 4.4.2 packages (“GSVA”, “ComplexHeatmap”, “corrplot”, “fmsb” and “ggplot2”).

### 2.8 Study approval

The study was approved by the Comite de Ética em Pesquisa do Hospital Universitário Cassiano Antonio de Morais in Brazil and the Rutgers Biomedical Health Sciences Institutional Review Board in Newark, NJ. Written informed consent was obtained from all participants as per the consent procedure approved by IRBs from all participating institutions.

## 3 Results

### 3.1 . T-SCM, T-CM, T-TM and T-EM memory CD4^+^ T cell subsets FACS-purified from individuals with LTBI demonstrate distinct Mtb antigen-induced transcriptional programs

**Figure 1:**
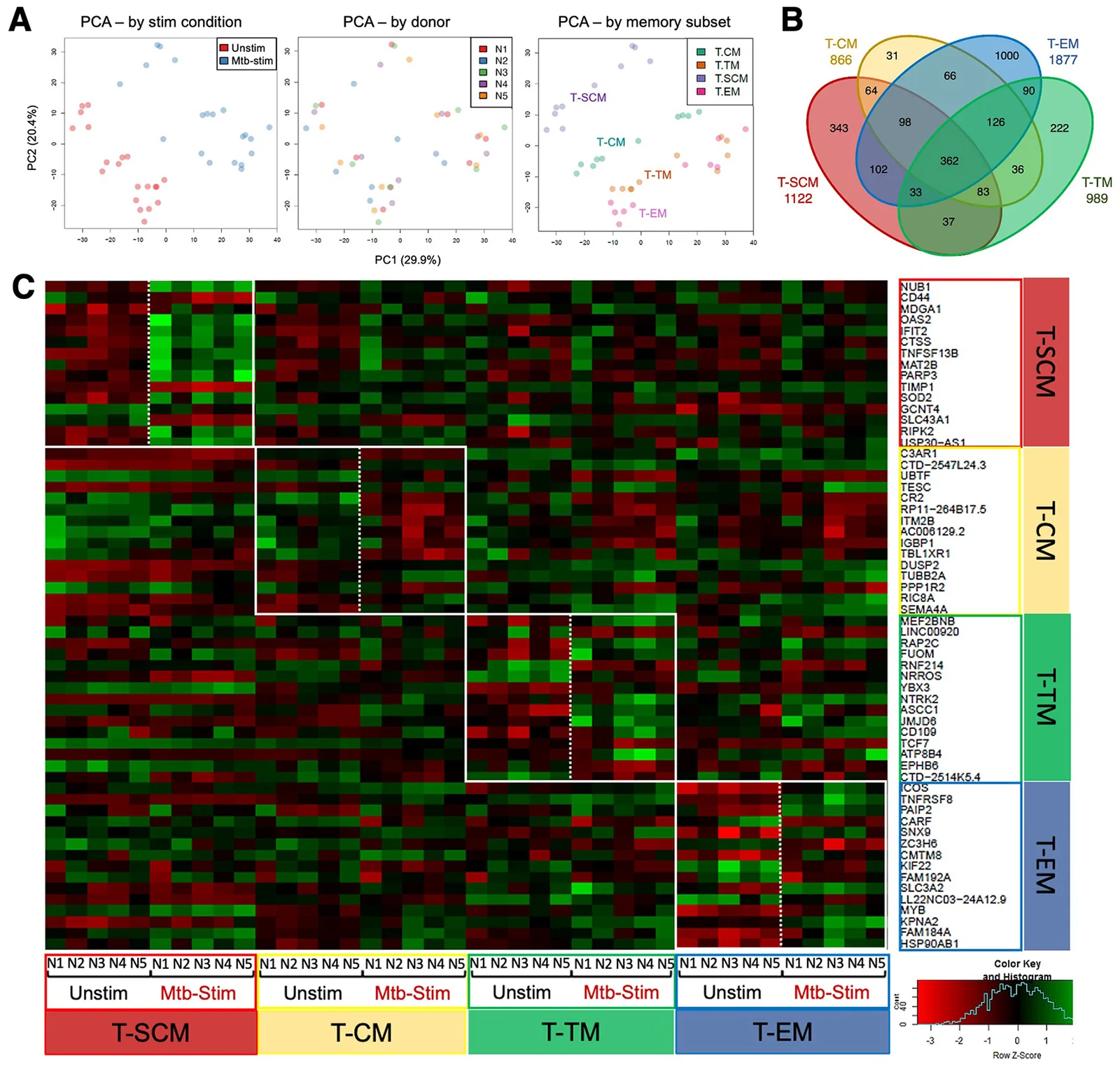
Mtb antigen-specific stimulation induces differential gene expression patterns across CD4^+^ memory T cell subsets. RNA-seq analysis of sorted T-memory subsets from PBMC of donors with remote LTBI (Brazil, *n* = 5) identified 4,170 total DEG (adjusted *p*-value cutoff < 0.001) with Mtb-antigens (Mtb-stim) or co-stimulation-only (Co-stim) samples. **(A)** PCA plots showing clustering based on stimulation condition, donor, and T-memory subset. **(B)** Venn diagram representing overlap of DEGs between the global gene list and each of the four subset-specific genes (*n* = 5 Mtb-stim, *n* = 5 Co-stim-only for each cell type; adjusted *p*-value cutoff < 0.001). **(C)** Heatmap depicting expression of the top 15 DEGs for all subsets based on Mtb-stim and Co-stim conditions. PCA, principal component analysis; T-SCM, stem cell memory, T-CM, central memory; T-TM, transitional memory; T-EM, effector memory.

PBMC from five donors with remote LTBI (Brazil) were cultured for 16 h with co-stimulatory antibodies (anti-CD28 and anti-CD49d), with or without Mtb antigens ESAT-6 and CFP-10 (“Mtb-stim” or “Co-stim-only”, respectively). CD4^+^ T memory cell subsets were identified as stem cell memory (T-SCM): CD45RA^+^CD45RO^−^CCR7^+^CD27^+^CD95^+^; central memory (T-CM): CD45RA^−^CD45RO^+^CCR7^+^CD27^+^CD95^+^; transitional memory (T-TM): CD45RA^−^CD45RO^+^CCR7^−^CD27^+^CD95^+^; and effector memory (T-EM): CD45RA^−^CD45RO^+^CCR7^−^CD27^−^CD95^+^. These subsets were subsequently purified by fluorescence-activated cell sorting (Supplementary Figure S1 and Supplementary Table S1) and analyzed by RNA sequencing. A total of 4,170 DEGs were identified. Principal component analysis (PCA) further demonstrated a clear segregation by stimulation condition and did not vary between individuals (Figure 1A). However, memory subsets clustered in a graded manner, with T-SCM most distinct and T-TM/T-EM grouping closely (Figure 1A). DEG analysis further revealed subset-specific signatures and 362 shared genes (Figure 1B). To visualize a representative snapshot of the most robust subset-specific shifts, we identified the top 15 DEGs upon Mtb-stimulation within each subset (Figure 1C).

Notably, the cell surface adhesion and migration receptor *CD44* was downregulated in T-SCM across all individuals, whereas cell cycle and energy metabolism-associated enzyme-encoding genes, poly (ADP-ribosyl) transferase 3 (*PARP3*), methionine adenosyltransferase (*MAT2B)* (37, 38), and the interferon-induced enzyme oligoadenylate synthetase 2 (*OAS2)* (39) were upregulated in response to Mtb-stim (Figure 1C). T-CM showed down-regulated complement cascade receptors *C3AR1* and *CR2*, and elevated expression of cytoskeletal organization genes beta-tubulin 2A (*TUBB2A)* and the microtubule formation regulator GTPase *RIC8A* (40). On the other hand, T-TM up-regulated BLOC-1 Related Complex Subunit 8 (*MEF2BNB*/*BORCS8)* known to play a role in lysosomal delivery to phagosomes (41) and T-EM upregulated the T-cell co-stimulatory molecule TNF receptor superfamily member 8 (TNFRSF8/CD30) (42), indicating the activated effector status of these subsets (Figure 1C).

### 3.2 . Mtb antigen-responsive CD4^+^ T memory subsets exhibit distinctive immunometabolism-associated gene expression profiles

**Figure 2:**
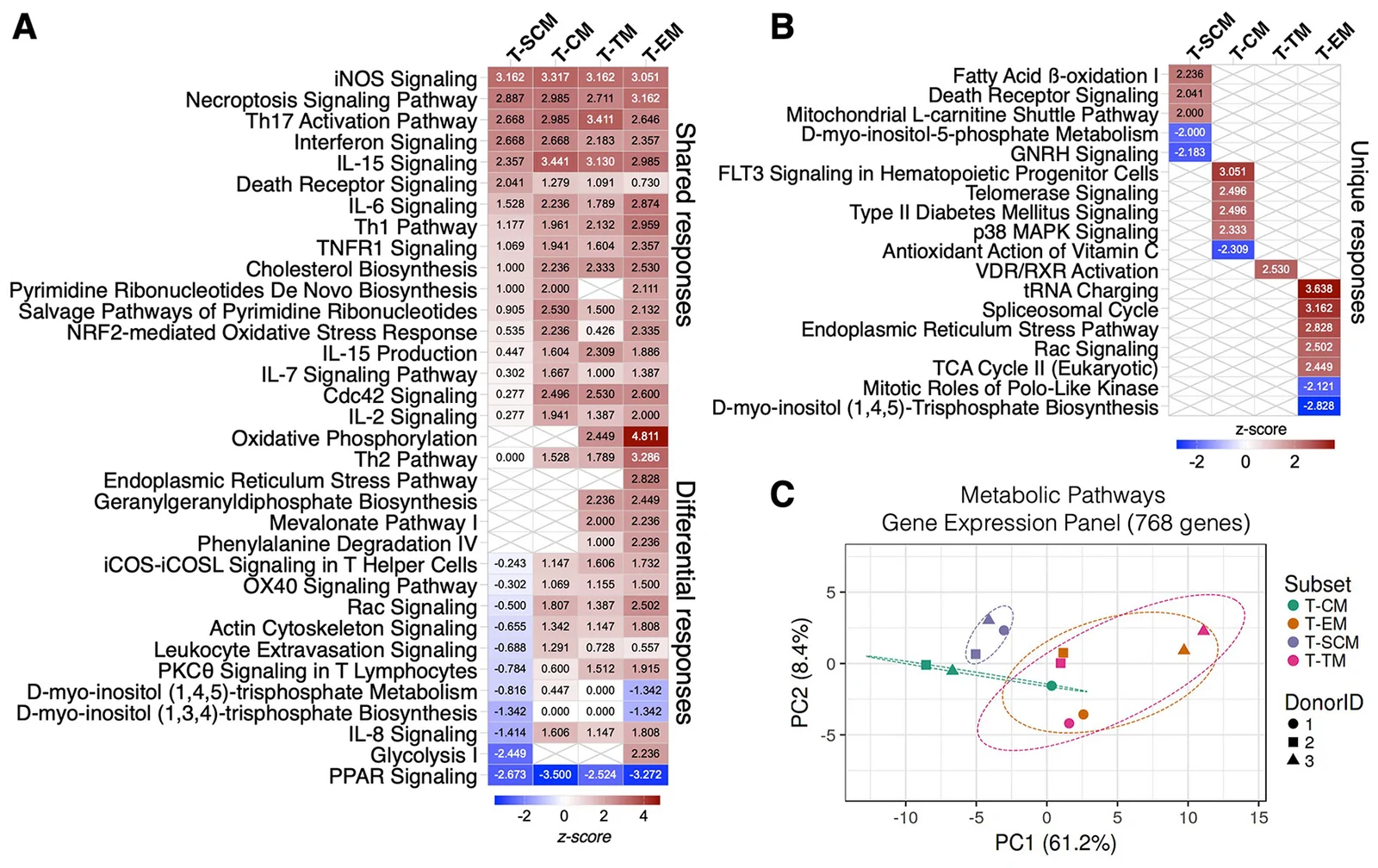
CD4^+^ memory T cell subsets display shared, differential, and unique pathways in response to Mtb antigens. PBMC from donors with remote LTBI (Brazil, *n* = 5) were stimulated with Mtb-antigens for 16 h and analyzed via RNA-sequencing. Ingenuity Pathway Comparison Analysis was performed to identify pathways (filtered by *p* < 0.01) to depict: **(A)** Heatmap of predicted activation (red) or inhibition (blue) of canonical pathways displaying shared and differential responses across memory cell subsets; **(B)** Heatmap of canonical pathways displaying unique responses across cell subsets; **(A, B)** z-score of 0 represents an enriched pathway with no predicted directional bias. “X” denotes pathways that were not detected at the statistical significance threshold. **(C)** Cross-platform metabolic validation: PCA plot showing clustering of memory subsets from a subset of these donors from Brazil (*n* = 3), based on normalized mRNA counts of 768 metabolism-associated genes quantified using the Human Metabolic Pathways Panel (NanoString), in response to Mtb-stimulation. PCA, principal component analysis; T-SCM, stem cell memory; T-CM, central memory; T-TM, transitional memory; T-EM, effector memory.

Pathway-level comparison analysis of the RNA-seq data (Brazil, *n* = 5 donors) using the Ingenuity Pathway Analysis software identified subset-specific shared and differential responses (Figure 2A) and unique responses (Figure 2B). Across all subsets, interferon signaling, nucleotide biosynthesis, and multiple cytokine pathways (iNOS, Th17, Th1, IL-6, IL-2, IL-15) were activated, while PPAR signaling, a regulator of lipid metabolism, inflammation, and differentiation, was suppressed (43) (Figure 2A). T-CM, T-TM and T-EM subsets showed strong enrichment of OX40 signaling, a co-stimulatory pathway critical for T cell activation (44) (Figure 2A). Notably, T-SCM cells exhibited reduced leukocyte extravasation, PKCθ signaling, IL-8 signaling, and glycolysis compared to other subsets (Figure 2A), yet uniquely upregulated fatty acid oxidation (FAO) and mitochondrial metabolism (Figure 2B), a profile consistent with their identity as a long-lived, self-renewing reservoir rather than immediate effector activity. The enhanced FAO in T-SCM was not accompanied by increased OXPHOS, suggesting diversion of citrate to cytosol (carnitine-shuttle pathway), a process that may sustain self-renewal while reducing serum fatty acids (45, 46). Compared to T-SCM cells, T-CM cells exhibited higher cytokine- and leukocyte extravasation signaling, alongside enrichment in FLT3 and telomerase signaling, suggesting both effector priming and self-renewal capacity (Figure 2B). T-TM upregulated VDR/RXR signaling (Figure 2B), which has been linked to the production of the antimicrobial peptide cathelicidin (47). Finally, T-EM cells displayed the highest glycolytic activity among the four subsets (Figure 2A), alongside TCA metabolism and cell cycle programs including Rac family small GTPase signaling (Figure 2B), previously implicated in cytoskeletal spreading, polarization, and chemotaxis occurring during T cell activation (48).

A targeted validation of the RNA-seq data using 768 metabolism-associated genes (NanoString's Human Metabolic Pathways gene expression panel) confirmed a separation among memory subsets based on metabolism-associated genes alone (Figure 2C). Gene Ontology analysis (Supplementary Figure S2) further confirmed that T-SCM and T-CM subsets expressed proliferative and oxidative pathways, including aerobic respiration, nucleotide synthesis, purine metabolism, and ATP turnover. T-CM additionally upregulated phosphorus metabolism processes, potentially reflecting phosphate-sensitive signaling pathways previously shown to support CD8^+^ T-cell-mediated clearance of Mtb (49). In contrast, T-TM cells showed enrichment of antimicrobial responses and protein stabilization pathways, while T-EM cells were characterized by elevated glycolysis, pyruvate metabolism, nucleotide phosphorylation, and ADP-to-ATP cycling, indicating heightened cellular activation and signal transduction. Collectively, these data further substantiate that CD4^+^ memory T cell subsets activate unique immunometabolic pathways in their recall response to Mtb stimulation.

### 3.3 . Met-Flow analysis identifies key differences in protein expression dynamics among CD4^+^ T memory subsets

Next, we identified overlapping immunometabolic signatures expressed across the four CD4^+^ memory subsets by comparing results of transcriptomic analyses via RNA-seq and NanoString platforms (Table 1). Notably, modules related to cell proliferation, mitochondrial metabolism, glucose uptake, pentose phosphate pathway activity, glycolysis, and antimicrobial function were consistently detected across both assays and emerged as differentially regulated among subsets at 16 h post stimulation with Mtb antigens.

**Table 1:** Overall enriched pathways associated with immunometabolism in CD4^+^ T memory subsets.

Results	T-SCM	T-CM	T-TM	T-EM
RNA-seq (DEGs and IPA; Figures 1, 2)	Cell cycle genes, FAO, Mitochondrial L-carnitine shuttle pathway	Cytoskeletal organization genes, Higher cytokine & extravasation signaling than T-SCM, Telomerase signaling, FLT3 signaling, p38 MAPK signaling	Lysosomal signaling genes, VDR/RXR signaling, OXPHOS	Activation marker genes, Glycolysis, ER stress, TCA cycle, OXPHOS high
NanoString RNA metabolism assay (GO pathways; Supplementary Figure S2)	Generation of precursor metabolites & ATP, Nucleotide metabolism, Purine metabolism	Aerobic respiration, Generation of precursor metabolites and ATP, Nucleotide metabolism, Purine metabolism, Phosphorus metabolism	Protein stability, Protein translation, Antimicrobial processes (antigen presentation, cell killing)	Glycolysis, Pyruvate metabolism, Nucleotide phosphorylation, ATP from ADP cycling
Overall enriched modules	• Mitochondrial metabolism • Fatty acid oxidation • Proliferation	• Mitochondrial metabolism • Proliferation and survival signaling • Effector-like activation (higher phosphorus metabolism reflects intermediates that regulate cytokine signaling and translation)	• Pentose Phosphate Pathway (production of ribose-5-phosphate & redox action supporting nucleotide biosynthesis, cytokine gene expression) • Glucose uptake • Antimicrobial activity	• Pentose Phosphate Pathway (produces ribose-5-phosphate, a precursor for nucleotide biosynthesis and precedes glycolysis) • Glucose uptake • Glycolysis • Antimicrobial activity

Table presents integrated findings from transcriptomic profiling via RNA-seq and NanoString assays.

Overall enriched immunometabolic modules were identified within each subset based on concordant signals across results from both platforms.

T-SCM, stem cell memory; T-CM, central memory; T-TM, transitional memory; T-EM, effector memory.

**Figure 3:**
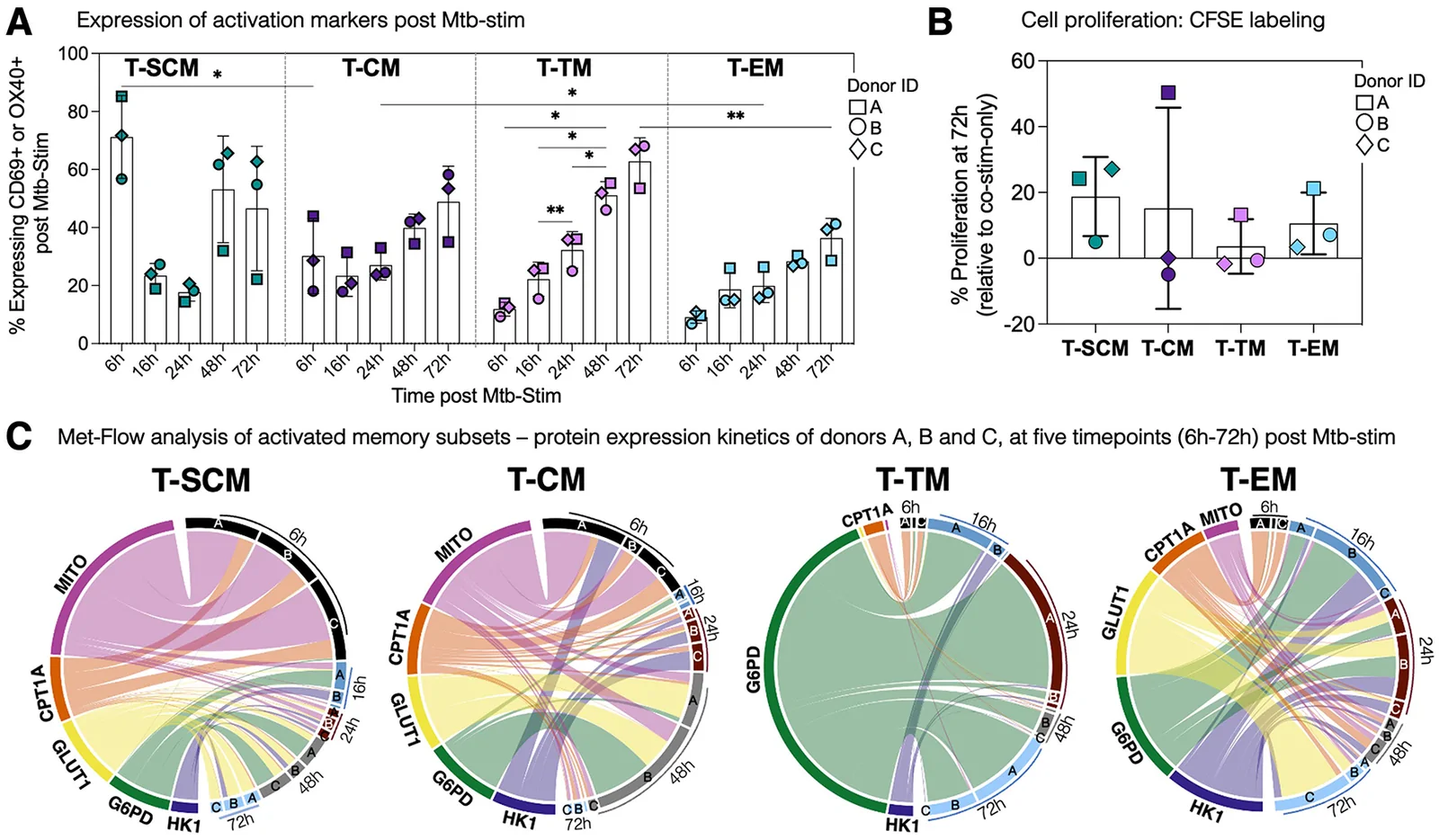
Cell proliferation and Met-Flow analysis of CD4^+^ T memory subsets. PBMC from three donors (A, B and C) with remote LTBI (North America) were stimulated with Mtb antigens (Mtb-stim), and readouts were quantified via flow cytometry at various timepoints 6-, 16-, 24-, 48- and 72-h post Mtb antigens stimulation: **(A)** Activation: bar graph showing expression of activation markers (depicted as percentage of cells expressing markers relative to respective donor's co-stimulation-only condition); **(B)** Cell proliferation: percent cell proliferation at 72 h quantified via CFSE dye labeling; and **(C)** Met-Flow analysis: GMFI values of metabolic protein marker expression in memory subsets, depicted in chord plots. Chord ribbons indicate linkage between markers (left) with timepoints and respective donors (right), and width of ribbons represents strength of GMFI. All data are represented as relative to each donor's respective co-stimulation-only condition. Significance was determined using repeated measures two-way ANOVA, with Tukey's multiple comparisons testing: *p < 0.03 and **p < 0.002, and only significant comparisons are shown. T-SCM, stem cell memory; T-CM, central memory; T-TM, transitional memory; T-EM, effector memory; GMFI, geometric mean fluorescence intensity.

To further evaluate temporal shifts in pathway utilization and functional expression, we extended our analysis to monitor changes in immunometabolic activity over five timepoints, at the protein level, in an independent cohort from North America (*n* = 3). We utilized the Met-Flow assay (22) using a panel comprising markers of memory subsets, activation, proliferation, effector cytokines and five metabolic proteins and rate-limiting enzymes representative of key biochemical pathways: CPT1A (Fatty-acid oxidation), G6PD (Pentose Phosphate Pathway), GLUT1 (Glucose transport), HK1 (Glycolysis) and Mitotracker (mitochondrial activation & ATP biosynthesis; Supplementary Table S2). Relative to the co-stimulation-only condition, all Mtb-stimulated memory subsets demonstrated an increase in the expression of activation markers CD69 or OX40 (CD134) over time (Figure 3A). T-SCM cells demonstrated an overall trend of higher cell proliferation at 72 h across all donors than T-TM and T-EM (Figure 3B).

We then compared the expression of metabolic proteins by activated memory T cell subsets, across five timepoints, based on the expression of OX40 or CD69 as a surrogate of Mtb antigen-driven activation upon stimulation (Figure 3C and Supplementary Figure S3). Interestingly, across all donors, T-SCM exhibited high intensity (GMFI) of mitochondrial activity and CPT1A expression as early as 6 h post Mtb-stimulation, and a gradual shift toward G6PD and GLUT1 expression by 48 h and 72 h (Figure 3C, Supplementary Figure S3A). T-CM had a similar profile to that of T-SCM but displayed lower mitochondrial activity and higher HK1 expression than T-SCM. In contrast, T-TM were characterized by CPT1A and HK1 expression at 6 h and 16 h and a strong co-expression of G6PD at 16 h and at all other timepoints. Similarly, T-EM showed an overall lower mitochondrial activity with CPT1A expression at 6 h, followed by high G6PD, HK1 and GLUT1 expression at other timepoints (Figure 3C and Supplementary Figure S3A). Of note, Mtb-stimulation induced a higher frequency of T-SCM positive for G6PD and HK, yet these cells maintained a lower metabolic intensity than T-CM, T-TM or T-EM (Supplementary Figures S3A, B). Taken together, these findings further validate the early transcriptional modules we observed and identify ensuant shifts in the metabolism of Mtb-specific memory T cell subsets: T-SCM rely on early mitochondrial metabolism and sustained regulation, T-CM maintain a balanced oxidative-glycolytic profile, and T-TM and T-EM rapidly engage the pentose phosphate pathway and glycolysis.

### 3.4 . CD4^+^ T-TM and T-EM subsets display enhanced anti-mycobacterial effector functions

**Figure 4:**
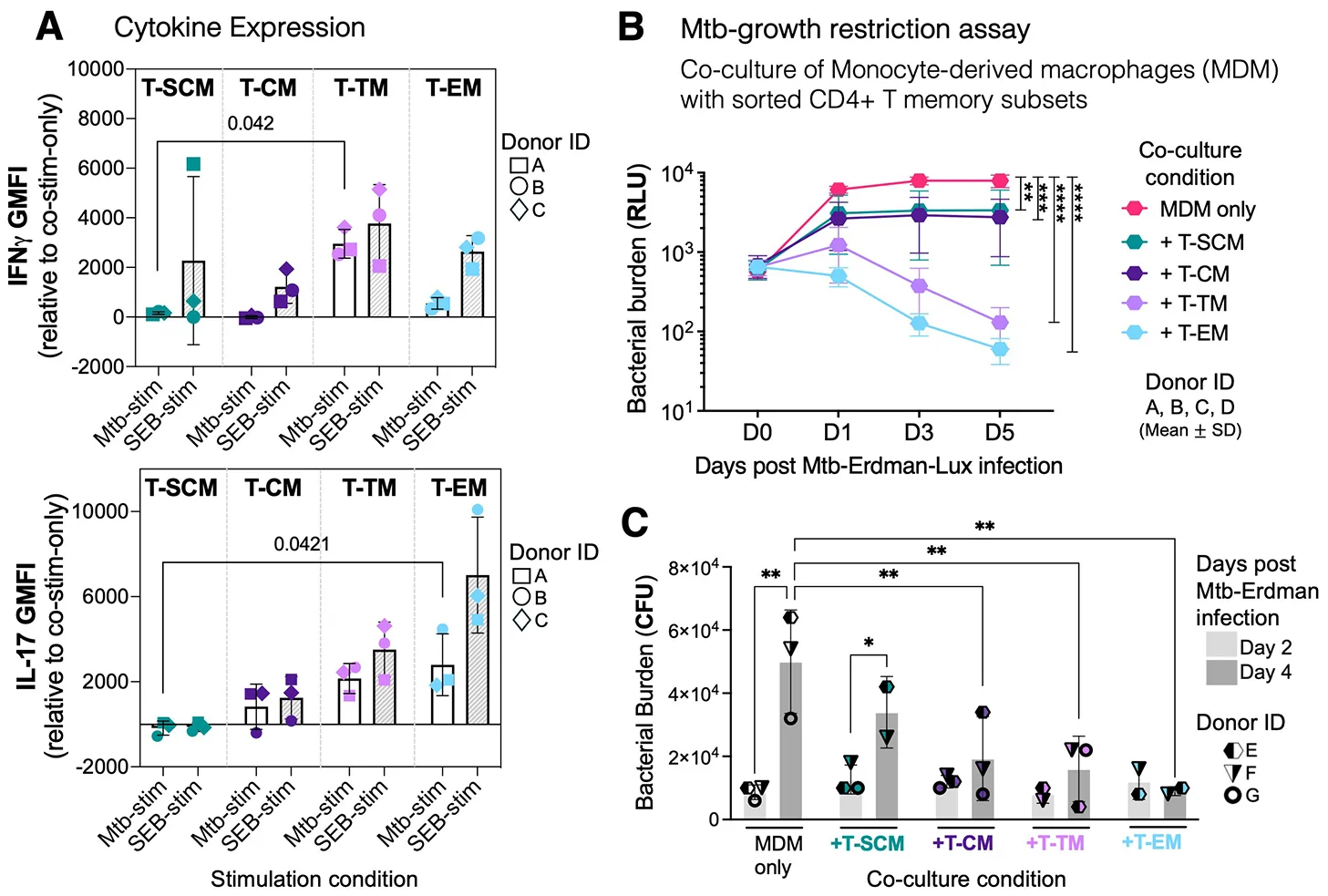
Comparison of anti-mycobacterial effector responses of CD4^+^ T memory subsets. **(A)** Functional mapping of recall responses: PBMC from donors **(A–C)** with remote LTBI (*n* = 3, North America) were stimulated with Mtb antigens (Mtb-stim) for 16 h and subjected to intracellular staining and flow cytometry analysis. Bar plots illustrate the expression intensity (GMFI) for IFNγ+ (top) and IL-17+ (bottom) activated CD4+ CD69/OX40+ T memory subsets. Data are normalized to donor-matched co-stimulation-only conditions. **(B)** Intracellular Mtb growth restriction assay: monocyte-derived macrophages (MDM) from an independent cohort of donors with remote LTBI (*n* = 4, North America) were infected with luciferase-expressing Mtb Erdman (5 MOI) and co-cultured with FACS-sorted autologous memory T cell subsets. Bacterial burden was longitudinally monitored via luminescence (RLU, top) at days 0, 1, 3, and 5 post-infection. **(C)** Validation of antimicrobial activity: Intracellular Mtb growth restriction was quantified via Colony Forming Units (CFU) in another independent donor cohort with remote LTBI (*n* = 3, North America). Cell lysates were plated on days 2 and 4 post-infection on 7H11 agar plates and enumerated. Data **(B, C)** are expressed as Mean with SD. Significance was determined using two-way ANOVA, with Tukey's multiple comparisons testing: *p < 0.03 and **p < 0.002. GMFI, geometric mean fluorescence intensity; FACS, fluorescence associated cell sorting; RLU, relative luminescence units; MOI, multiplicity of infection; T-SCM, stem cell memory; T-CM, central memory; T-TM, transitional memory; T-EM, effector memory.

Next, we determined whether the distinct metabolic changes occurring in the four subsets associated with subsequent differences in anti-mycobacterial effector functions. Specifically, we evaluated the levels of cytokines IFNγ and IL-17, which have been well-described to mediate potent protective immune response cascades against Mtb (50). Following stimulation with either Mtb-antigens or superantigen staphylococcal enterotoxin B (SEB) (non-specific activation positive control), PBMC (*n* = 3, North America) were analyzed by flow cytometry to quantify the geometric mean fluorescence intensity (GMFI; Figure 4A) and frequency (%) (Supplementary Figure S4A) of IFNγ and IL-17 expressing cells. We observed that T-TM and T-EM subsets exhibited a significantly higher magnitude of Mtb-specific cytokine expression when compared with T-CM and T-SCM. Although T-SCM and T-CM had higher frequencies of IL-17-producing cells, their GMFI magnitude was lower (Supplementary Figure S4B). In contrast, IFNγ responses in these subsets were reduced in both frequency and magnitude. T-TM cells displayed the highest frequency and magnitude of IFNγ production, indicating enhanced effector function in more differentiated memory subsets (Figure 4A, Supplementary Figures S4A, B).

To evaluate an additional functional readout beyond cytokine expression, we performed an Mtb growth restriction assay to assess the ability of the four memory subsets to limit intracellular bacterial growth (Figure 4B). First, monocyte-derived macrophages (MDM) were purified and cultured from PBMC of donors with LTBI (*n* = 4, North America) and infected with luciferase-expressing Mtb-Erdman (5 MOI). Infected MDM were then co-cultured with each of the four memory T cell subsets (1:1 ratio), which were purified via 4-way-FACS-sorting, from the respective donors (Supplementary Figure S1). Bacterial burden [quantified as relative luminescence units (RLU)] was quantified at days 1, 3 and 5 post *in-vitro* infection. By days 3 and 5 post-infection, MDM alone had significantly higher bacterial burden in the absence of any memory T cell subsets (Figure 4B). However, MDM-T memory cell co-culture showed a graded effector response, with higher Mtb growth restriction capability of T-TM and T-EM subsets than T-SCM and T-CM (Figure 4B). These results were further confirmed in an independent cohort of donors (*n* = 3, North America), by plating intracellular Mtb on 7H11 agar media and quantifying colony forming units (CFU) on days 2 and 4 post-infection (Figure 4C).

### 3.5 . Baseline blood samples from non-progressors revealed a distinct “stem-cell-like” immunometabolic gene signature compared to progressors

**Figure 5:**
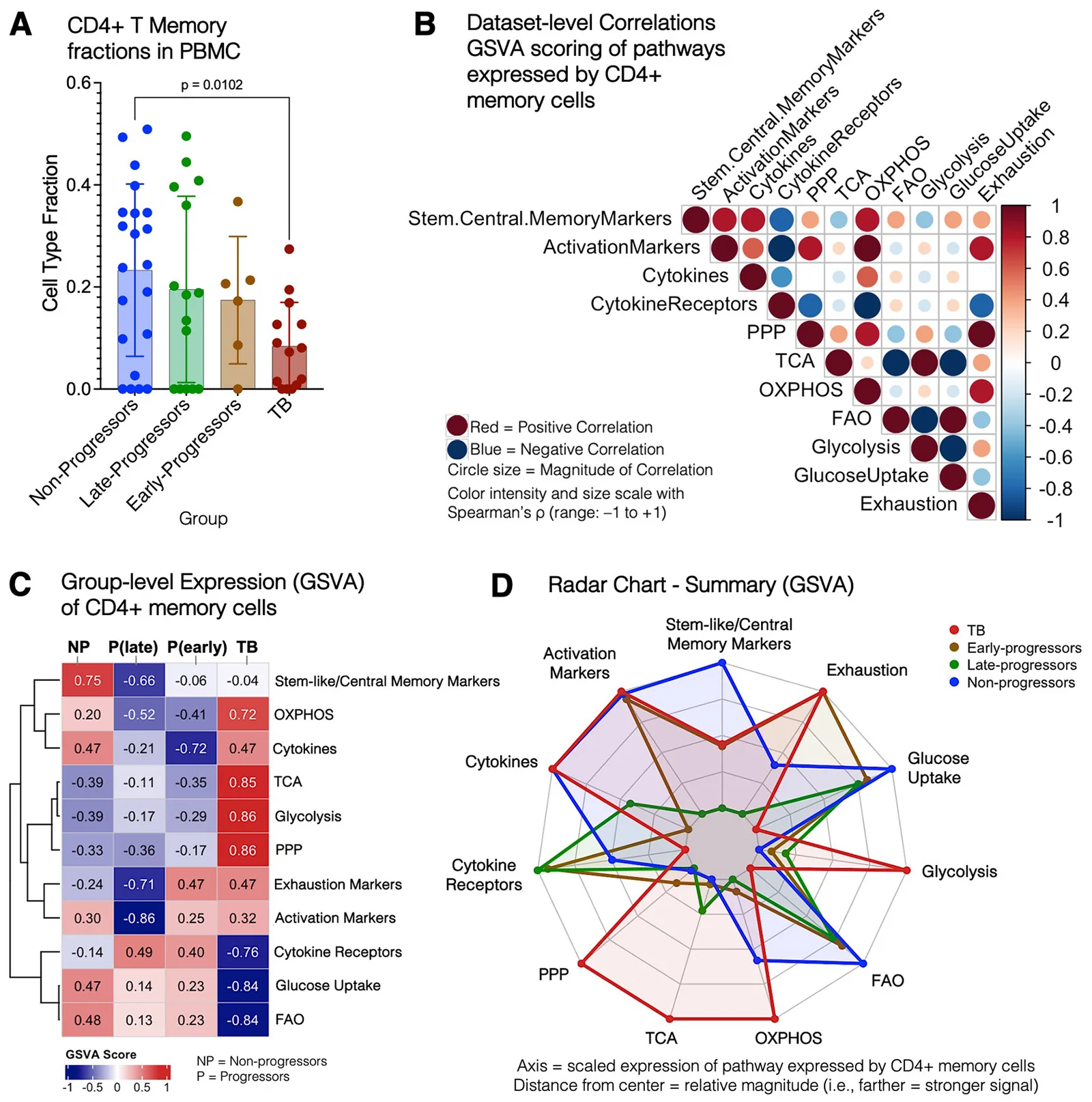
Cellular deconvolution analysis of an independent GSE112104 cohort of TB progressors, non-progressors and TB cases. RNAseq data of baseline, unstimulated PBMC samples from 16 Progressors and 21 Non-Progressors (post initial exposure to index case) and 14 individuals with TB was used for cellular deconvolution analysis using the CIBERSORTx software (*p* < 0.05, 1,000 iterations). “Early” progressors developed TB within 102 days, while “Late” progressors developed TB between 103–1,795 days post study enrollment. Total CD4^+^ T memory cells were compared between all groups: **(A)** bar graph showing cell fractions in PBMC, significance was determined using Welch ANOVA and Browne-Forsythe, with Dunnett's T3 multiple comparisons test and significant comparisons with *p*-values (<0.05) are indicated; **(B)** dataset-level correlations of inferred immunometabolic pathways (scored with GSVA, and scaled with Spearman's rho [range −1 to 1, positive (red), negative (blue)]); **(C)** relative group-level expression patterns of pathways scored with GSVA; and **(D)** spider plot depicting a summary of overall pattern of expression and association with inferred pathways among the groups (GSVA), distance away from center represents increasing magnitude of expression. NP, non-progressors; *P*, progressors.

To evaluate whether immunometabolic states observed in antigen-responsive CD4^+^ memory subsets from remote LTBI donors were evident in a larger, external cohort with longitudinally defined TB outcomes, we analyzed and inferred the baseline gene expression from bulk RNA-seq data of our previously published PBMC dataset (GSE112104) from Brazil, comprising TB cases (*n* = 14) and Mtb-infected individuals who eventually progressed to TB (“progressors”, *n* = 16) and those that did not progress and remained asymptomatic (“non-progressors”, *n* = 21) at clinical follow-up (51). Progressors were stratified by time to disease onset: “early” ( ≤ 102 days) and “late” (103–1,795 days). CIBERSORTx cellular deconvolution analysis (35) revealed that non-progressors had higher proportions of total CD4^+^ memory cells at baseline (Figure 5A, Supplementary Figure S5).

**Figure 6:**
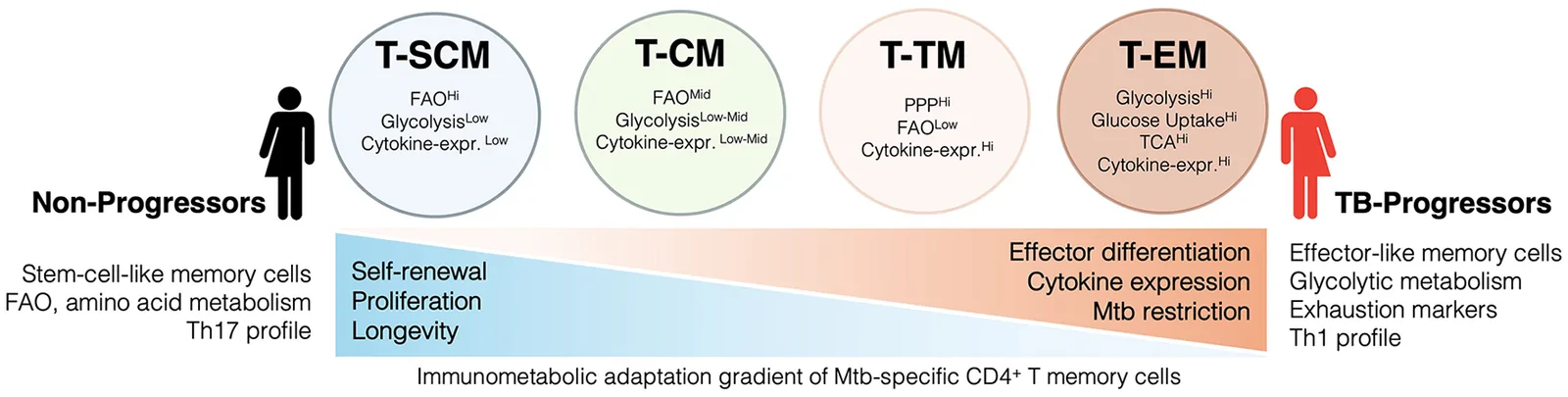
Summary of the immunometabolic adaptation gradient of Mtb-specific CD4^+^ T memory cells. Schematic illustrating the progressive differentiation and metabolic reprogramming of Mtb-specific CD4^+^ memory T cell subsets, spanning from stem-like T-SCM to T-CM to highly functional T-TM and T-EM states. Each subset is characterized by distinct metabolic signatures, ranging from FAO dominance in T-SCM and T-CM, to elevated glycolysis, TCA activity, and glucose uptake in T-EM. T-TM cells exhibited high PPP activity and lower FAO. The accompanying arrows below denote the shift from self-renewal and longevity (blue) toward terminal differentiation and antimicrobial activity (red). Cytokine expression increased along the gradient, culminating in robust effector function and Mtb growth restriction by T-EM cells. Individuals from Brazil who progressed to active TB (progressors) exhibited a predominance of effector-like CD4^+^ memory cells with a Th1-skewed cytokine profile and expression of exhaustion markers. In contrast, non-progressors retained stem-like and FAO-enriched subsets with a Th17-like profile, consistent with enhanced metabolic fitness and long-term immune control.

Next, we curated gene sets representing key metabolic pathways, stem-cell-like/central memory markers, activation and exhaustion markers, cytokines, and cytokine receptors for comparison across CD4^+^ memory cells from all groups (Supplementary Table S3). Global expression of stem-like/central memory markers in the GSE112104 dataset correlated positively with mitochondrial metabolism and activation and inversely with glycolysis (Figure 5B), consistent with our prior findings. CD4^+^ memory cells from non-progressors showed the highest stem-cell-like and FAO signatures, while TB cases exhibited elevated glycolysis and PPP activity, indicating a shift toward a glycolytic, pro-inflammatory state (Figures 5C, D). TB cases also showed high OXPHOS, TCA metabolism and upregulation of exhaustion markers, likely due to increased biosynthetic demands from systemic inflammation and sustained immune activation. Interestingly, early-progressors expressed higher activation, PPP, cytokine and exhaustion scores than late-progressors, suggesting an effector-like, transitional immunometabolic phenotype (Figures 5C, D). Overall, progressors showed increased *HK1, ATP5A1, GLUT1*, and *IFNG* expression, whereas non-progressors expressed higher *CPT1A* and *IL17C* (Supplementary Figure S6). Comparison analysis of other pathways further revealed that progressors upregulated cytokine, integrin, eicosanoid, RAR, and apoptosis signaling, while non-progressors favored mitochondrial metabolism and amino acid signaling (Supplementary Figure S6). Together, these observed shifts in pathway enrichment suggest that specific metabolic states underpin functional immune CD4^+^ memory cell phenotypes and associate with disease progression trajectory (Figure 6). An integrated sample map and experimental platforms utilized in this study have been provided in Supplementary Table S4.

## 4 Discussion

Understanding long-term immune control in LTBI, driven by memory CD4^+^ T cells, is vital to inform strategies to prevent progression to active disease. In this study, we characterized distinct immunometabolic signatures associated with recall responses of Mtb antigen-responsive CD4^+^ memory T cell subsets in individuals with remote LTBI across independent cohorts from Brazil and North America, with cross-platform validations. Our results support a model of progressive differentiation within the CD4^+^ memory T cell compartment and reveal how discrete metabolic programs may shape functional specialization and protective capacity.

Resting memory T cells rely on mitochondrial metabolism including oxidative phosphorylation (OXPHOS) and fatty acid oxidation (FAO), but upon antigen re-challenge, utilize glycolysis to support differentiation into effector T cells (52). Long-lived CD8^+^ memory T cells have been reported to exhibit higher mitochondrial mass and fused morphology, favoring respiration, while the fragmented mitochondria in effector subsets that generate more reactive oxygen species (53–55) have a higher NADPH requirement to mitigate oxidative stress (56). Consistent with these reported findings, the Met-Flow analysis showed elevated and sustained mitochondrial activity in T-SCM. We also observed elevated frequencies of G6PD and HK1-expressing T-SCM cells with low GMFI, suggesting that T-SCM may act as metabolic reservoirs, utilizing a “low-burn” strategy that prioritizes mitochondrial regulation and FAO over the high-magnitude biosynthetic bursts seen in T-TM and T-EM. This restrained metabolic profile likely preserves their long-term survival and “stem-like” potential during chronic Mtb exposure. T-CM shared a similar profile to that of T-SCM, but produced higher IFNγ and IL-17, indicating a more differentiated phenotype than T-SCM. In contrast, T-TM and T-EM subsets exhibited features of effector polarization including higher HK1 and G6PD expression, robust cytokine output, and enhanced Mtb restriction. These shifts align with known roles of G6PD and the pentose phosphate pathway (PPP) in supplying NADPH to buffer oxidative stress and sustain cytokine production during ongoing infection (57–59). Together, these patterns suggest that metabolic programming not only reflects T cell fate but may actively shape antimicrobial function.

In cellular deconvolution analysis of PBMC collected post initial Mtb exposure to index TB cases in Brazil [RNA-seq dataset GSE112104 (51)], memory CD4^+^ T cells from progressors and TB cases exhibited T-TM/T-EM-like effector differentiation, higher exhaustion markers, elevated *IFNG* expression and strong signatures of glycolysis and PPP activity. In contrast, non-progressors showed an activated stem-cell-like/central memory cell phenotype marked by elevated FAO, reduced glycolytic activity and lower *IFNG* levels, consistent with reports linking FAO-derived citrate to HK1 inhibition (46, 60) and dampened IFNγ production (61). Instead, non-progressors expressed higher *IL17C*, corresponding with recent studies linking higher IL-17 levels and Th17-like T cells in non-progressors than progressors (25, 62, 63) and with other studies describing Th17-mediated protection in multiple models of TB (50, 64–66). Higher circulating numbers of Mtb-specific Th17-like CD4^+^CCR6^+^CXCR3^+^CCR4^−^ memory cells have also been reported in individuals with LTBI (17, 67, 68), leading us to speculate that acquisition of IFNγ- or IL-17-driven effector function during memory development may ultimately impact long-term Mtb control. Moreover, while both Th1 and Th17 cells depend on glycolysis, Th17 cells exhibit a greater reliance on amino acid metabolism, particularly glutaminolysis (69, 70). Notably, non-progressors displayed upregulation of proline biosynthesis, choline catabolism, glutamine metabolism and apoptosis inhibition, suggesting metabolically adaptive Th17-like memory cells favoring survival, homeostasis, and long-term persistence (21).

The metabolic immunomodulation of memory T cells may maintain Mtb in its latency, preventing progression to active disease. However, this endogenous control may not equate to sterilizing immunity, as memory cells are tuned to the antigenic load and environment of the initial infection. For instance, larger loads of viable or replicating Mtb may skew memory T cell differentiation toward PPP- and glycolysis-dependent, short-lived effector phenotypes with increased proinflammatory cytokine production (18). These cells may exhibit reduced longevity and become prone to functional exhaustion (71), leading to insufficient bacterial control and progression to TB. On the other hand, given their defining ability to self-renew and differentiate into T-CM, T-TM and T-EM memory subsets, T-SCM cells represent a foundational component of durable immune memory and functional resilience.

Recent studies have highlighted the therapeutic potential of targeting metabolic checkpoints to bolster memory T cell function: modulating PPP and glycolytic flux to enhance cytokine production, and promoting FAO to support T cell longevity and self-renewal [reviewed in (72)]. Thus, therapies targeting metabolic pathways to favor FAO, could restore or sustain the functional capacity of CD4^+^ memory T cells in TB progressors, particularly in T-SCM and T-CM subsets. Notably, the transitional metabolic features observed in late-progressors suggest a window of plasticity that may be amenable to such immunomodulation. Future longitudinal cohort studies can determine if metabolic exhaustion precedes or follows the failure of bacterial containment and allow for more precise targeting of the immunomodulators.

Because our study focused on PBMC samples from a limited number of donors with remote LTBI, we acknowledge that such peripheral sampling may not capture the complexity of antigen-specific vs. bystander activated cells and tissue-resident memory populations, or account for confounders such as persistent TST/IGRA positivity vs. reversion, Mtb re-exposure, comorbidities, and concurrent infections. To address these limitations, we tracked donor-to-donor variability in our analyses and complemented subset-resolved experimental data with transcriptomic inference from the larger, independent GSE112104 cohort. We recognize that these memory CD4^+^ signatures likely reflect cumulative immune activation from diverse inflammatory exposures and may not be exclusively Mtb-specific. Accordingly, this validation analysis was not designed to assign transcriptomic states to individual CD4^+^ memory subsets at single-cell resolution, but rather to assess whether the immunometabolic programs identified in antigen-responsive memory subsets from donors with remote LTBI are recapitulated at the population level across distinct clinical outcomes.

Remarkably, the enrichment of FAO-associated, stem-cell-like metabolic signatures in non-progressors, contrasted with glycolysis- and PPP-dominated effector-like programs in progressors and TB cases, mirroring the CD4^+^ memory subset-level metabolic spectrum we observed experimentally, i.e., from T-SCM/T-CM to T-TM/T-EM following Mtb antigen recall. This convergence across independent datasets suggests that immunometabolic specialization within the CD4^+^ memory compartment, rather than the absolute presence of a single subset, may influence durability of immune control vs. progression to disease. In this context, metabolic pathways such as FAO, glycolysis, and PPP emerge as functional correlates of memory quality that could be leveraged both as biomarkers of progression risk and as targets for future vaccine strategies aimed at preferentially expanding the ratio of long-lived, self-renewing CD4^+^ memory T cells rather than short-lived effector populations.

Prior studies have examined the phenotypic markers and cytokine potential of naïve CD4^+^ T memory cells, TCM and TEM subsets during recent infection, LTBI and active TB disease (26, 73). We extend this work by integrating CD4^+^ memory subset-level immunometabolic profiling with functional Mtb restriction assays and independent transcriptomic analyses across TB progression outcomes, identifying metabolic state as a key axis linking memory T cell differentiation, restimulation-induced transcriptional programming, and durability of immune control. Together, data from this proof-of-concept study provide transcriptomic and functional evidence to inform future validation studies aimed at selectively expanding protective CD4^+^ memory subsets through immunomodulatory or vaccination strategies.

## Data Availability

The datasets presented in this study can be found here: GEO Accession number: GSE112918 https://www.ncbi.nlm.nih.gov/geo/query/acc.cgi?acc=GSE112918.
