# Supplementary material for: Immunometabolic reprogramming of Mycobacterium tuberculosis-responsive memory CD4+ T cell subsets is linked to long-term protective immunity
Source: Front Tuberc. Author manuscript; Available in PMC 2026 Jun 26. (PMC13299550; doi:10.3389/ftubr.2026.1783887)
Supplement: Supplementary File 1 [file NIHMS2185510-supplement-Supplementary_File_1.docx]

**SUPPLEMENTARY DATA TABLES AND FIGURES**

**Immunometabolic reprogramming of Mycobacterium tuberculosis-responsive memory CD4⁺ T cell subsets is linked to long-term protective immunity.**

Authors and affiliations:

Vaishnavi Kaipilyawar^1^^[[1]](#footnote-1)^*, Samantha Leong^1^^[[2]](#footnote-2)^*, Arianne Lovey^1^, Lorenzzo L. Stringari^2^, Reynaldo Dietze^2^, Jerrold J. Ellner^1^, Rodrigo Ribeiro-Rodrigues^2,3^ and Padmini Salgame^1†^

^1^Department of Medicine, Center for Emerging Pathogens, Rutgers New Jersey Medical School, Newark, NJ, USA

^2^Núcleo de Doenças Infecciosas, Universidade Federal do Espirito Santo, Vitória, Brazil

^3^Laboratório Central de Saúde Pública do Estado do Espírito Santo (LACEN-ES), Secretaria de Saúde do Estado do Espírito Santo, Vitória, Espírito Santo, Brazil.

^†^Corresponding author:

Padmini Salgame, PhD

Rutgers New Jersey Medical School

Public Health Research Institute

225 Warren St, W250H, Newark, New Jersey, 07102, USA

+1-973-972-8647

[salgampa@njms.rutgers.edu](mailto:salgampa@njms.rutgers.edu)

**SUPPLEMENTARY DATA - TABLES**

| **Donor ID (N#) and Stimulation Condition** | **T-SCM** | **T-CM** | **T-TM** | **T-EM** |
| --- | --- | --- | --- | --- |
| N1 CD4 Co-stim-only | 87836 | 318320 | 96998 | 126463 |
| N1 CD4 Mtb-stim | 284502 | 389316 | 111634 | 158429 |
| N2 CD4 Co-stim-only | 39936 | 194893 | 72423 | 26168 |
| N2 CD4 Mtb-stim | 97758 | 267014 | 73340 | 28859 |
| N3 CD4 Co-stim-only | 57658 | 234083 | 6490 | 13210 |
| N3 CD4 Mtb-stim | 198274 | 248950 | 6863 | 13732 |
| N4 CD4 Co-stim-only | 160223 | 359291 | 31112 | 16055 |
| N4 CD4 Mtb-stim | 285486 | 445686 | 33132 | 16472 |
| N5 CD4 Co-stim-only | 16369 | 124498 | 14763 | 16424 |
| N5 CD4 Mtb-stim | 27283 | 141746 | 9870 | 13041 |

**Supplementary Table 1. Sorted CD4^+^ memory T cell counts obtained.** Table representing the final count of live, singlet CD4+ T cell subsets recovered upon FACS cell sorting (strategy outlined in Fig S1) and subjected to RNA-sequencing analysis.

| **Marker** | **Marker Type** | **Primary pathway** |
| --- | --- | --- |
| OX40/CD134 | Activation | T cell co-stimulation |
| CD69 | Activation | Early activation marker |
| CFSE  (Carboxyfluorescein succinimidyl ester) | Cell proliferation | Dye covalently binds intracellularly to cells and daughter cells have half the amount of dye with each division |
| GLUT1  (Glucose Transporter Type 1) | Metabolism | Glucose uptake/import |
| G6PD  (Glucose-6-Phosphate Dehydrogenase) | Metabolism | Pentose phosphate pathway |
| CPT1A  (Carnitine palmitoyltransferase 1A) | Metabolism | Fatty acid oxidation |
| HK1  (Hexokinase 1) | Metabolism | Glycolysis |
| Mitotracker  (MitoTracker™ Red CMXRos, ThermoFisher) | Metabolism | Mitochondrial activity ATP biosynthesis |

**Supplementary Table 2. Activation, proliferation and metabolism markers assayed for flow cytometry profiling.** Table depicting activation markers, cell proliferation, and metabolism markers, and their associated pathways, used for evaluating immunometabolic status of Mtb-stimulated CD4^+^ memory subsets using flow cytometry.

**Supplementary Table 3. Curated gene sets representing key metabolic pathways and immune activation used for GSVA.** Table depicting a custom list of genes that were grouped into biologically relevant categories reflecting immune phenotypes and metabolic pathway activity. Stem-cell-like/Central Memory Markers include genes associated with T-SCM/T-CM cells. Activation Markers represent surface and signaling molecules upregulated during T cell activation. T cell exhaustion is represented by exhaustion markers. Cytokines and Cytokine Receptors encompass effector molecules and their corresponding signaling receptors. Metabolic categories include the Pentose Phosphate Pathway, TCA Cycle, Oxidative Phosphorylation, Fatty Acid Oxidation, Glycolysis, and Glucose Uptake, each defined by canonical enzymes and transporters involved in energy production, biosynthesis, and nutrient sensing. These gene sets were used subsequently to interpret immunometabolic correlations and pathway-level enrichment using Gene Set Enrichment Analysis (GSVA).

| **Cohort** | **Platform** | **Donor ID** | **Location** | **Primary Assay** | **Figure Reference** |
| --- | --- | --- | --- | --- | --- |
| Discovery | RNA-Seq | N1, N2, N3, N4, N5  (n=5) | Brazil | Global Transcriptomics (IPA) | Fig 1,  Fig 2A-2B |
| Transcriptomic Validation | NanoString | N1, N2, N3  (n=3) | Brazil | Metabolic Gene Panel (768 genes) | Fig 2C,  Fig S2 |
| Functional Phenotyping | Flow Cytometry | A, B, C  (n=3) | North America | Activation Markers & Cytokine Expression | Fig 3  Fig S3A-B  Fig 4A  Fig S4A-B |
| Functional  Effector Response | MDM-T cell subset  co-culture | A, B, C, D  (n=4) | North America | Mtb bioluminescence (RLU) | Fig 4B |
| Functional  Effector Response Validation | MDM-T cell subset  co-culture | E, F, G  (n=3) | North America | Mtb colonies enumeration  (CFU) | Fig 4C |
| Independent HHC Cohort with known Clinical Outcomes | Public RNA-seq dataset  (External cohort) | Non-Progressors (n=21);  Progressors (n=16);  TB (n=14) | Brazil | Cellular Deconvolution, GSVA and Pathway Analysis | Fig 5  Fig S5  Fig S6 |

**Supplementary Table 4. Integrated sample map and experimental platforms.**

This table outlines the multi-platform approach used to characterize the metabolic and functional landscape of CD4+ memory T cell subsets. Samples were derived independent donor cohorts to ensure the geographic and biological reproducibility of our findings. Abbreviations: IPA= Ingenuity Pathway Analysis, MDM= monocyte-derived macrophages, RLU= relative luminescence units, CFU= colony forming units, GSVA= gene set variation analysis, TB= tuberculosis.

**
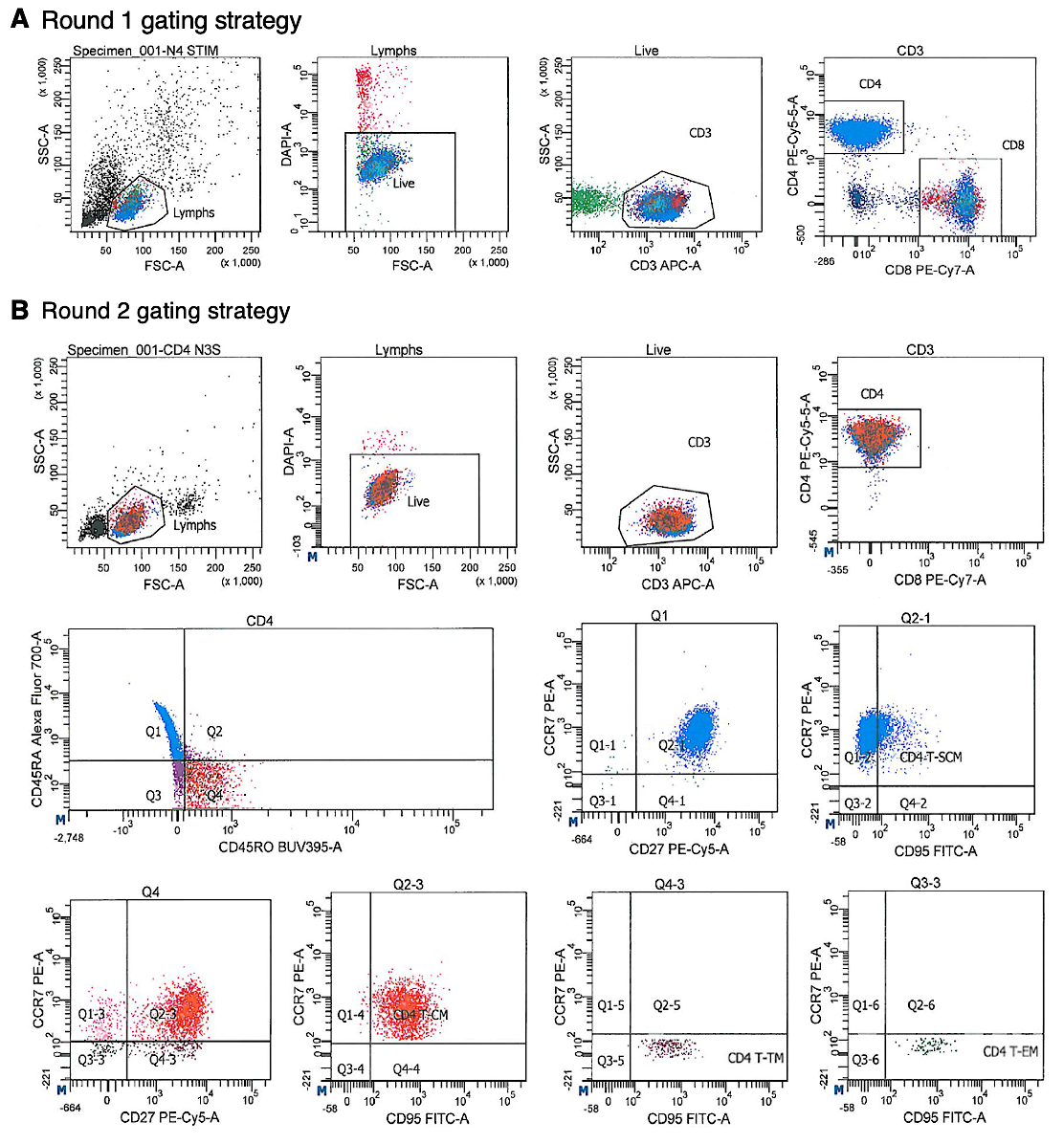
SUPPLEMENTARY DATA - FIGURES**

**Supplementary Figure S1**. **Gating strategies used for CD4^+^ memory T cell sorting.** (A) Round 1 sorting strategy of total stained PBMC to isolate Live (DAPI-negative), CD3^+^, CD4^+^ lymphocytes. (B) Round 2 sorting strategy of Round 1 sorted Live, CD4^+^ T cells to isolate the following four memory subsets: stem cell memory T cells (T-SCM): CD45RA^+^CD45RO^-^CCR7^+^CD27^+^CD95^+^; central memory T cells (T-CM): CD45RA^-^CD45RO^+^CCR7^+^CD27^+^CD95^+^; transitional memory T cells (T-TM): CD45RA^-^CD45RO^+^CCR7^-^CD27^+^CD95^+^; and effector memory T cells (T-EM): CD45RA^-^CD45RO^+^CCR7^-^CD27^-^CD95^+^.
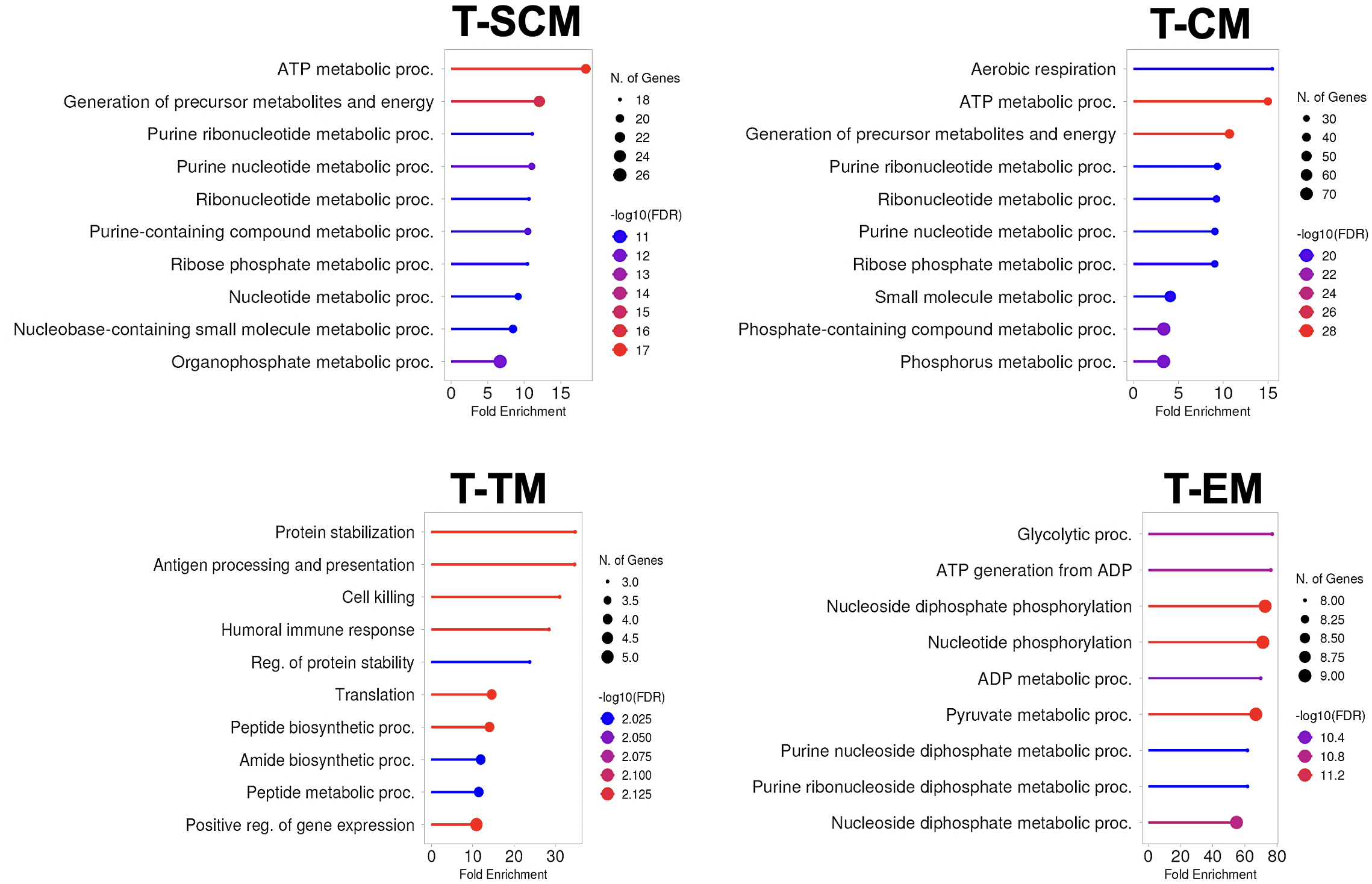


**Supplementary Figure S2. Pathway Analysis of metabolism genes quantified using NanoString assay.** Gene ontology analysis of biological process (ShinyGO 0.82) showing fold enrichment of genes and associated pathways in the four CD4^+^ T memory subsets, with FDR-cutoff 0.05 and minimum pathway size of 200. Gene expression was quantified using the NanoString Human Metabolism Panel comprising 768 genes, normalized and filtered for high expressing gene counts (log2 fold change >2.5), averaged across 3 individual donors (Donors N1-N3, Brazil). Abbreviations: T-SCM= stem cell memory. T-CM= central memory. T-TM= transitional memory. T-EM= effector memory.

**
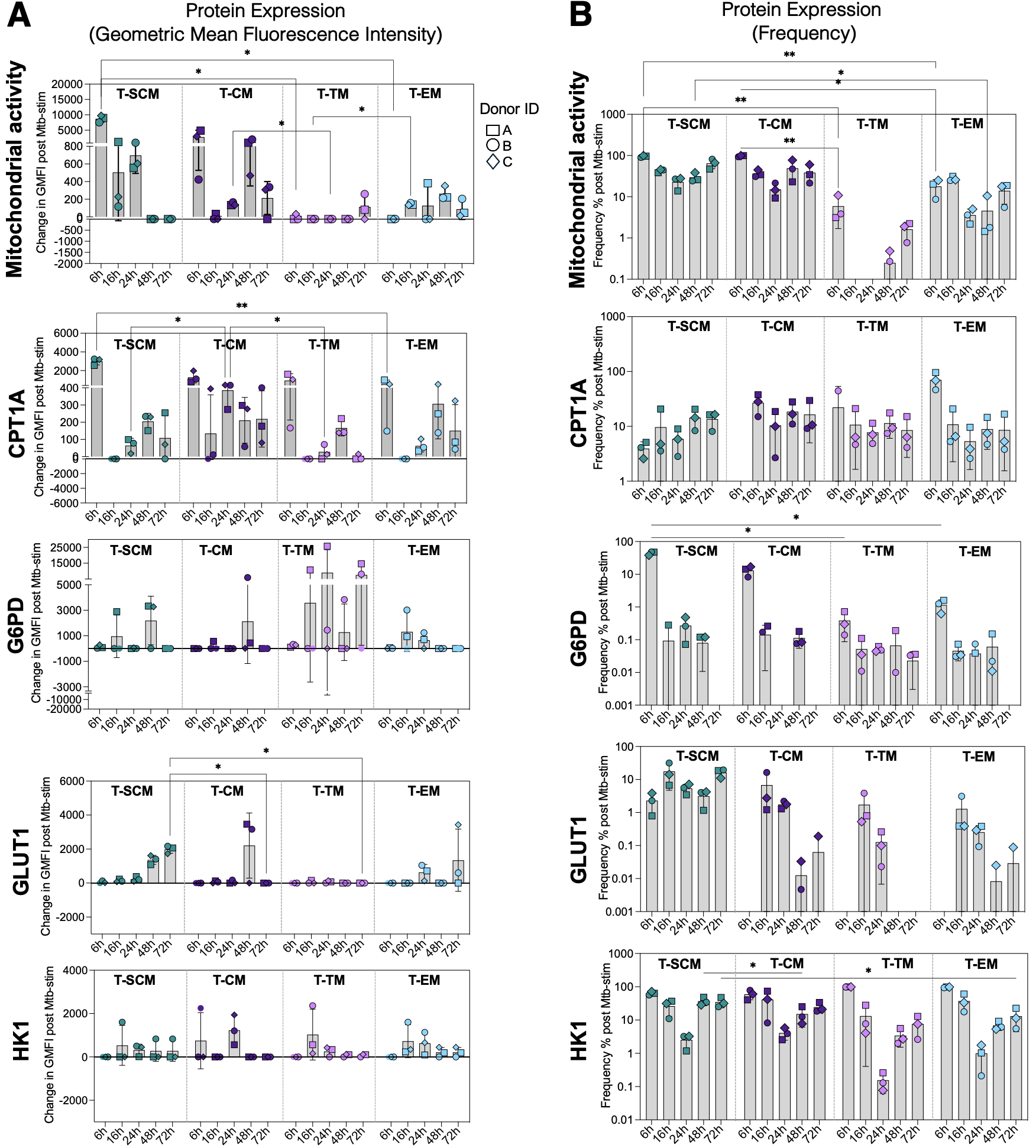
**

**Supplementary Figure S3. Quantification of metabolism proteins (representative of key biochemical pathways) from CD4+ T memory subsets, using Met-Flow assay.** (A) Expression Intensity (GMFI) and (B) Frequency (%) values of five metabolism markers in the four memory T cell subsets quantified at 6-,16-,24-,48- and 72h-post Mtb-stimulation, via the Met-Flow cytometry assay. GMFI data are presented as change in GMFI post Mtb-stim, relative to co-stimulation-only condition and expressed as Mean with SD for three donors A, B and C, from North America. Significance determined via repeated measures two-way ANOVA with Tukey’s test for multiple comparisons: *p<0.03, **p<0.002. Only significant comparisons across timepoints are shown between subsets. Abbreviations: GMFI= geometric mean fluorescence intensity, T-SCM= stem cell memory. T-CM= central memory. T-TM= transitional memory. T-EM= effector memory.


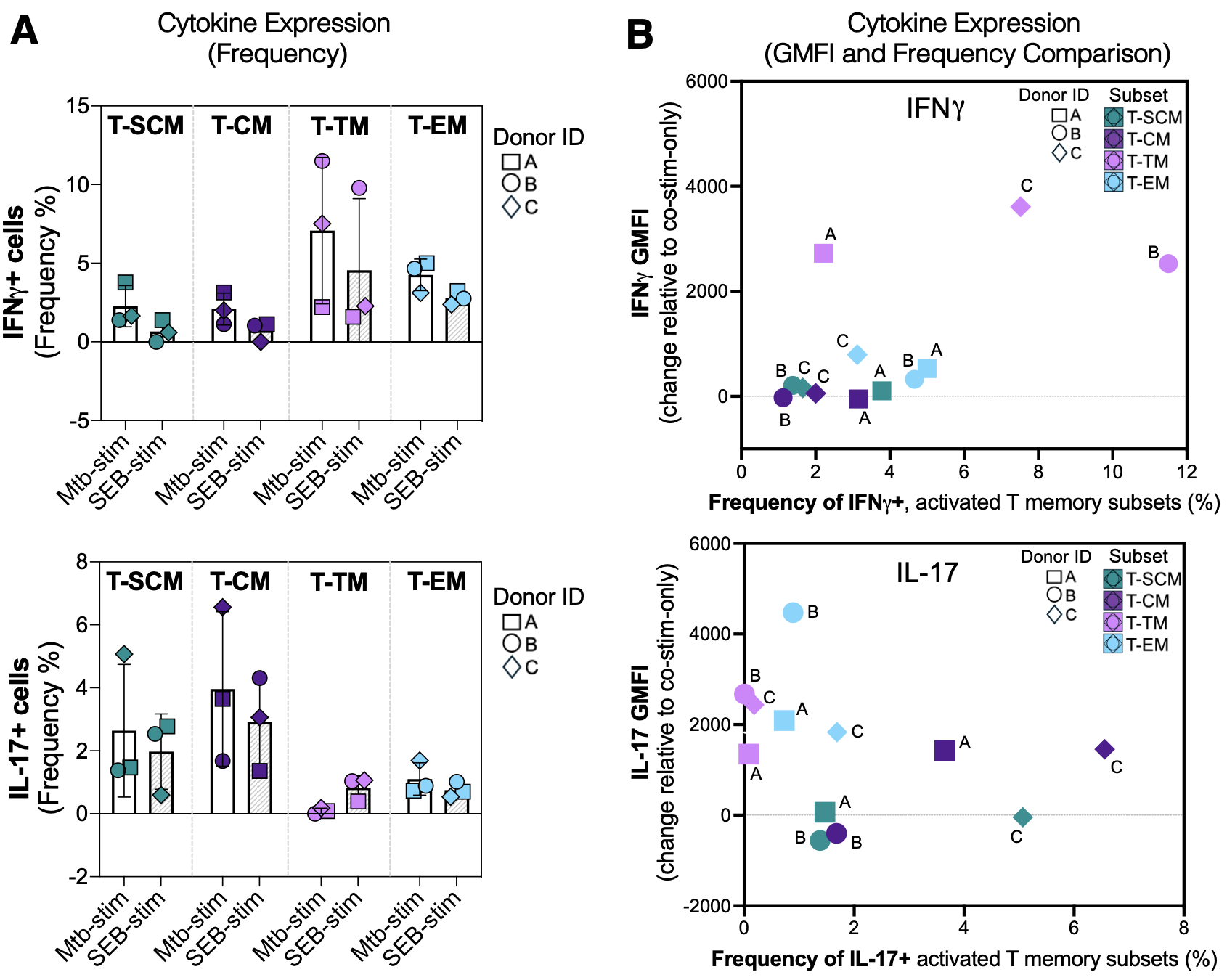


**Supplementary Figure S4. Quantification of Mtb antigen-induced cytokine expression by CD4+ T memory subsets.** PBMC from donors with remote LTBI (n=3, donors A, B and C, North America) were stimulated with Mtb antigens (Mtb-stim) or SEB (SEB-stim) for 16h and subjected to intracellular staining and flow cytometry analysis. (A) Bar graphs of cytokine expression are presented as frequency (%). (B) Comparison between frequency and GMFI: Scatter plots of GMFI (Y-axis) and frequency (X-axis) for IFNγ+ (top) and IL-17+ (bottom) for activated CD69/OX40+ T memory subsets. Significance was determined using two-way ANOVA with Bonferroni correction, and alpha<0.05 for multiple comparisons testing. No significant comparisons p<0.05 were found. Abbreviations: SEB= staphylococcal enterotoxin B, GMFI = geometric mean fluorescence intensity, T-SCM= stem cell memory, T-CM= central memory, T-TM= transitional memory, T-EM= effector memory.

**
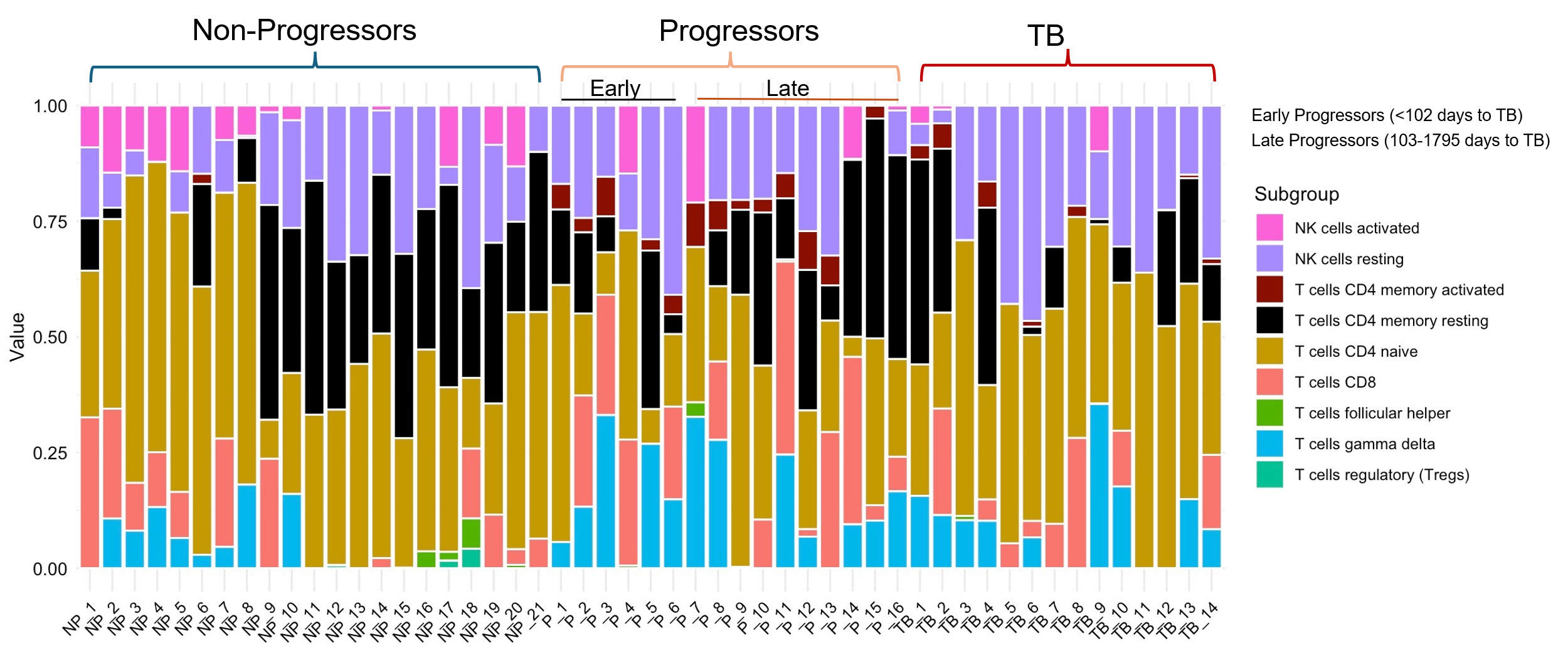
**

**Supplementary Figure S5**. **Cellular Deconvolution Analysis of GSE112104, public RNA-seq dataset (Leong et.al, 2020).** Bar plot depicting cell composition (value represents fraction) of baseline, unstimulated PBMC samples from 16 TB Progressors (6 “Early” and 10 “Late”) and 21 Non-Progressors (post initial exposure to index case), and 14 individuals with TB, using CIBERSORTx cellular deconvolution analysis (p<0.05, 1000 iterations).

**
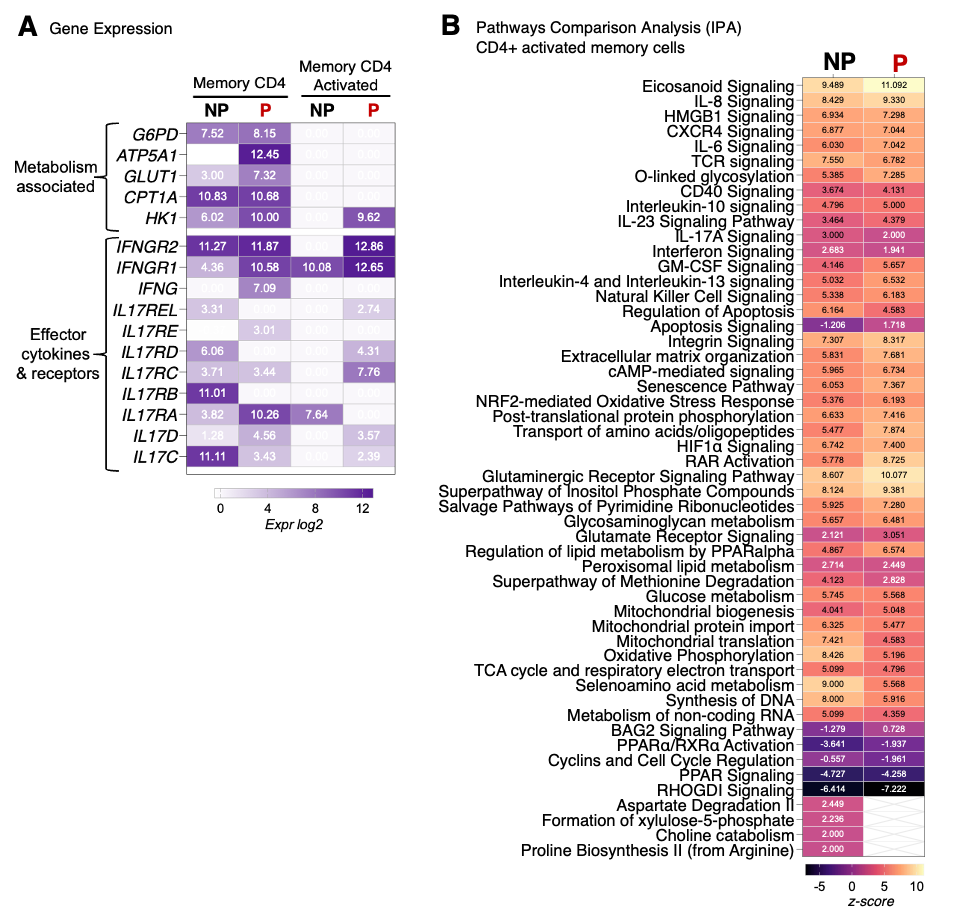
**

**Supplementary Figure S6. Cellular deconvolution-based gene expression and pathway analysis of activated CD4+ memory T cells in GSE112104, public RNA-seq dataset (Leong et.al, 2020).** Comparison of gene expression derived from PBMC deconvolution analysis of CD4^+^ IL-7R^+^ resting memory and CD4^+^ IL-7R^-ve^ CD44^hi^ activated memory cells in non-progressors and progressors**:** (A) log2 gene expression of metabolic markers, effector cytokines and receptors; and (B) canonical pathways among activated CD4+ subsets (IPA analysis, z-score ± 1.5 and p<0.001). z-score of 0 represents an enriched pathway with no predicted directional bias. "X" denotes pathways that were not detected at the statistical significance threshold. Abbreviations: NP=non-progressors, P=progressors.

1. * equal contribution [↑](#footnote-ref-1)
2. [↑](#footnote-ref-2)
